# Socioeconomic inequalities in vaccine uptake: A global umbrella review

**DOI:** 10.1371/journal.pone.0294688

**Published:** 2023-12-13

**Authors:** Amber Sacre, Clare Bambra, Josephine M. Wildman, Katie Thomson, Natalie Bennett, Sarah Sowden, Adam Todd

**Affiliations:** 1 Population Health Sciences Institute, Newcastle University, Newcastle, United Kingdom; 2 National Institute for Health and Care Research (NIHR) Applied Research Collaboration (ARC) North East and North Cumbria (NENC), Newcastle, United Kingdom; 3 ScotCen Social Research, Edinburgh, United Kingdom; 4 School of Pharmacy, Newcastle University, Newcastle, United Kingdom; University of Udine: Universita degli Studi di Udine, ITALY

## Abstract

This global umbrella review aimed to synthesise evidence of socioeconomic inequalities in the uptake of routine vaccinations and identify the mechanisms that may contribute to the association. To our knowledge, no attempt has been made to synthesise the global body of systematic reviews across a variety of vaccines, geographical locations, and measures of SES. The inclusion criteria were as follows: studies assessing vaccination uptake according to education, income, occupation/employment, and/or area-level deprivation; any country or universally recommended routine vaccination (according to the WHO); qualitative or quantitative reviews, published 2011-present. The searches were performed in eight databases. The screening process followed PRISMA-E guidelines, each stage was performed by one reviewer, and a 10% sample checked by a second for consistency. Included reviews underwent data extraction, quality appraisal (AMSTAR-2), and narrative synthesis according to country-context. After deduplication, 9,163 reports underwent title and abstract screening, leaving 119 full texts to be assessed for eligibility. Overall, 26 studies were included in the umbrella review. Evidence for lower uptake amongst disadvantaged SES individuals was found in all 26 reviews. However, 17 reviews showed mixed results, as inverse associations were also identified (lower uptake for advantaged SES, and/or higher uptake for disadvantaged SES). Those that explored high-income countries had a greater prevalence of mixed findings than those focusing on low/middle-income countries. The two most frequently cited mechanisms were vaccination knowledge, and confidence in vaccination or vaccination providers. These mechanisms were often understood by review authors as varying by level of education. We find socioeconomic differences in routine vaccination uptake, but the association did not always follow a gradient. Whilst education may be associated with uptake globally, our study indicates that its role varies by country-context. A limitation is the overlap of some primary studies across the included systematic reviews.

## Background

The Coronavirus pandemic has highlighted the stark socioeconomic inequalities in health outcomes and healthcare access [1, 2] and supposedly fuelled the “largest sustained decline in childhood vaccinations in approximately 30 years”, with 25 million children globally missing out [3]. Moreover, as a preventative form of healthcare, vaccination is essential in reducing the mortality and morbidity associated with vaccine preventable diseases [4]. For instance, there were an estimated 604,000 new cases of cervical cancer globally in 2020 and 342,000 deaths [5]. The human papillomavirus (HPV) is responsible for more than 95% of cervical cancer incidences [5], and is often transmitted through sexual contact, as the majority of HPV types reside in the mouth, throat or genital area [6]. Thus, achieving and maintaining high levels of vaccination uptake is widely beneficial, as disease incidences can be significantly reduced or even eradicated. Often, discussions of vaccine uptake refer to physical and non-physical factors that can either aid or hinder access to vaccination [7]. For instance, the SAGE Working Group on Vaccine Hesitancy produced the Vaccine Hesitancy Determinants Matrix, identifying three main groupings of factors that can potentially impact uptake: contextual influences, individual and group influences, and vaccine/vaccination-specific issues [7].

The already complex process of vaccination can be experienced differently by individuals based on wider social inequalities. Health inequalities can be defined as “systematic, socially produced (and therefore modifiable) and unfair”, with socioeconomic status (SES) showing the most stark differences within a given population” [8 p. 2]. Generally, disadvantaged groups who experience higher levels of deprivation, lower levels of education, and lower incomes, are more likely to suffer from poorer health outcomes [9]. These are often referred to as “socioeconomic inequalities”, “disparities”, or “inequities”, and can be measured in a variety of ways [8–10]. Indicators of SES include income, occupation, education, and occasionally, place of residence [9]. Indeed, socioeconomic differences in vaccination uptake can vary over space (between-groups and within-group differences, such as ethnicity, or gender), time (changes to healthcare systems/policies), context (administration methods, or country) and by vaccine (influenza, HPV, or Coronavirus) [11–19]. For instance, the SAGE Working Group on Vaccine Hesitancy produced the Vaccine Hesitancy Determinants Matrix, identifying three main groupings of factors that can potentially impact uptake: contextual influences, individual and group influences, and vaccine/vaccination-specific issues [7].

Understanding the association between socioeconomic status and uptake is especially pertinent when considering the recent global decline in childhood vaccination [3]. For instance, if uptake of the Measles, Mumps and Rubella (MMR) vaccination is below 90% coverage in a given population, the diseases can quickly spread [20]. A study conducted in Liverpool, UK, exploring the measles outbreak in 2012–13, identified that deprived neighbourhoods had the highest proportion of susceptible children, due to under immunisation [21]. Consequently, these pockets of low uptake can in turn exacerbate socioeconomic inequalities in disease burden [1]. To our knowledge, no attempt has been made to synthesise the global body of literature on socioeconomic inequalities and vaccination uptake, across a variety of vaccines, geographical locations, and measures of SES, at the level of an umbrella review. Collating the existing evidence on socioeconomic inequalities in vaccination uptake may help understand the recent decline, and whether these inequalities are linked. Moreover, the mechanisms by which measures of socioeconomic status are associated with healthcare uptake in general are often inadequately explored [22]. Indeed, this is evident in the field of vaccine uptake where existing literature typically fails to consider the mechanism(s) explaining why these associations may occur. Understanding this association is especially relevant for vaccination policy makers and providers in order to identify where the impact of socioeconomic inequalities are most stark, which this umbrella review aims to do on a large scale. Therefore, we will explore both the presence of inequalities in vaccination uptake and the possible explanations for these differences. This review, therefore, aimed to: **(1)** examine whether there are socioeconomic inequalities in vaccine uptake and summarize the contexts in which they exist; **(2)** to identify any mechanisms that could potentially explain these inequalities.

## Methods

Umbrella reviews are often performed to synthesise a large body of literature and are consequently broad in their scope, sometimes focusing on multiple interventions [23]. They aim to provide a clear and concise summarises to a given topic [23]. This review followed Preferred Reporting Items for Systematic Reviews and Meta-Analyses Equity extension (PRISMA-E) guidelines, developed for systematic reviews with an equity focus [24]. A completed PRISMA-E checklist can be viewed in the additional material, **S1 Appendix**. This review was registered with PROSPERO (CRD42022334223) and a full protocol has been published [25]. The methodology and research questions were established *a priori*, as follows:

**Primary:** Are there socioeconomic inequalities in vaccine uptake?
**Secondary:** What are the mechanisms identified to explain such socioeconomic inequalities in vaccine uptake?

### Selection criteria

The inclusion criteria were kept deliberately broad in order to identify all relevant reviews. The inclusion criteria were conceptualised using the PECOS (Population, Exposure, Comparison, Outcome, Study Design) framework, as follows:

**Population:** General populations, including demographic sub-populations. All countries.

**Exposure:** Advantaged SES, according to the following indicators: education, income, occupation/employment, and/or measures of area-level deprivation/poverty (e.g., the English Indices of Multiple Deprivation (IMD)).

**Comparison:** Disadvantaged SES, according to the following indicators: education, income, occupation/employment, and/or measures of area-level deprivation/poverty.

**Outcome:** Variation in the rate (uptake), or proportion of a target population (coverage), that have been vaccinated. Eligible vaccines were those labelled by the World Health Organization (WHO) as universally recommended routine vaccinations [26]: BCG (Tuberculosis), Hepatitis B (Hep B), Polio (IPV/OPV), DTP-containing (Diphtheria, Tetanus and Pertussis) vaccine, Haemophilus influenzae type b (Hib), Pneumococcal (conjugate) (PCV), Rotavirus, Measles, Rubella, and HPV. Studies focusing on influenza and Coronavirus were also eligible for inclusion to account for reviews published in response to the Coronavirus pandemic. Eligible measures of uptake or coverage were: initiation and/or completion of multi-dose vaccines or vaccination schedules as a whole (where uptake or coverage is measured by the initiation/completion of several different vaccines, some with and without multiple doses).

**Study Design:** Only systematic reviews that synthesised quantitative or qualitative studies were included. The quantitative reviews did not have to include a meta-analysis. A systematic review was classified as such if it met four of the following criteria, as outlined by the Database of Abstracts of Reviews of Effects (DARE) [27]:

1. Were inclusion/exclusion criteria reported?
2. Was the search adequate?
3. Were the included studies synthesised?
4. Was the quality of the included studies assessed?
5. Are sufficient details about the individual included studies presented?

There were no language restrictions, any potentially relevant abstracts and titles were translated using online tools. A publication date of 2011 –present day was applied. The WHO’s 2011 “Global Vaccine Action Plan 2011–2020” report outlined the updated guidance on improving vaccination uptake [28]. The publication of this report may capture vaccination policy changes relevant to the present day, and as the WHO is a global institution, it is relevant to all countries. Further details on the inclusion and exclusion criteria are outlined in the additional material, **S2 Appendix**.

### Search strategy and information sources

The search strategy was developed and piloted in Medline (via Ovid) to test its sensitivity in returning eight previously identified indicator systematic reviews [11–18]. After initial piloting, the following eight databases were searched during the period 03/09/2022-04/09/2022: Medline (via Ovid), Embase (via Ovid), Cumulated Index to Nursing and Allied Health Literature (CINAHL) (via EBSCO), Cochrane CENTRAL, Science Citation Index (SCI) (via Web of Science), Database of Abstract Reviews of Effects (DARE), Scopus (via Elsevier), and Applied Social Sciences Index and Abstracts (ASSIA) (via ProQuest). Grey literature searching was conducted using the WHO repositories and PROSPERO. The full search strategy can be seen in the additional material, **S3 Appendix**.

### Screening, and selection

After the searches, the records were downloaded into Rayyan [29] and deduplicated. Title and abstract screening were conducted by reviewer one (AS), and a 10% sample was checked by reviewer two (KT), in reference to the *a priori* established eligibility criteria. The full-text screening stage was also performed by reviewer one (AS) and a 10% sample was checked by reviewer two (KT). The process of the double-checking process is to ensure a consistency during screening, thus ensuring all relevant reviews are included and all ineligible reviews are excluded. If an agreement could not be achieved, a third reviewer was consulted (AT) to establish consensus. Both forwards and backwards citation searching was performed using Web of Science to supplement the search.

### Data extraction

To aid the data extraction process, an extraction template was designed, and the following information was extracted from each of the systematic reviews: bibliographical details (author, year of publication, title, DOI, abstract), study design (satisfaction of the DARE criteria, method of synthesis, number of included studies); search specificities (databases, date, restrictions); any information relating to PICOS (geographical location, population, vaccine/s, definition of uptake, measures of socioeconomic inequality); the main findings/conclusions relevant to the umbrella reviews’ research questions (uptake percentages (rates), counts, odds ratios etc.); any potential mechanisms or pathways that may help account for the socioeconomic differences in vaccine uptake, as identified in the systematic review. Additionally, the following information was extracted from the primary studies, if provided by the systematic review in which they were included: authors, year of publication, vaccine/s of focus, geographical location, population, measures of socioeconomic inequality, risk of bias/quality verdict, overall uptake of specified vaccine, and the main findings. The data extraction was performed by reviewer one (AS) and checked in full by reviewer two (KT) for accuracy; any disagreements were discussed with reviewer three (AT) who helped establish a consensus.

### Dealing with overlap

In umbrella reviews, there is a risk of primary study overlap [23]. To address this, a citation matrix was created, and the corrected coverage area (CCA) was subsequently calculated and reported [30]. A CCA value equal to, or greater than, 15% indicates high overlap of primary studies [30].

### Quality appraisal

A MeaSurement Tool to Assess systematic Reviews (AMSTAR-2) was used to assess the quality of the included systematic reviews. This tool was selected because it can be used to assess both randomized control trials and non-randomised studies of interventions [31]. The checklist is comprised of 16 questions, of which seven are considered “critical domains” (see Shea *et al*. for more information [31]). The responses to each of the questions (see additional material, **S4 Appendix**) were recorded in a spreadsheet and then inputted into the online AMSTAR-2 tool that produces either a “high”, “moderate”, “low”, or “critically low” verdict [32]. Failure to meet one, or more, of these “critical domains” automatically reduces the overall score to “low” or “critically low”. AMSTAR-2 is not intended to produce an overall score but to identify areas of potential methodological weakness.

### Narrative synthesis

This process of narrative synthesis was guided by the Synthesis Without Meta-analysis recommendations [33]. Firstly, a table including the author, year of publication, vaccine/s, definition of uptake, geographical location, population, number of primary studies, number of relevant primary studies, and the measures of socioeconomic inequalities, was created. The reviews were then summarized using descriptive statistics, according to vaccine (childhood/adolescent, HPV, influenza/pneumococcal, or all routine vaccinations), geographical location (high-income countries, or low/middle-income countries, according to the World Bank classification [34]), and by measures of socioeconomic status (education, income, employment, area-level measures, or unspecified).

The key findings of each review were then summarised in a matrix-style table, disaggregated by country economic status. The results were narratively synthesised also according to country economic status to account for differences in healthcare spending. The synthesis approach used a range of terms to explain the findings from the included reviews; definitions of these terms can be seen in **Table 1**.

**Table 1:** Classification of findings and their definitions.

Classification of Findings	Definition
**Inequalities (conventional)**	Advantaged SES, higher vaccination uptake, AND/OR disadvantaged SES, lower vaccination uptake. These associations are “conventional” because they reflect the conclusions of wider healthcare equity literature, as outlined in the introduction [10, 22].
**Inverse (unconventional)**	Disadvantaged SES, higher vaccination uptake, AND/OR advantaged SES, lower vaccination uptake. These associations are “unconventional” because they are not reflective of wider social inequalities, nor the healthcare equity literature [10, 22].
**Mixed**	Evidence of inequalities and inverse associations.
**Consistent**	There is evidence for the stated association (inequalities, inverse, or mixed) across all primary studies in the included systematic reviews.
**Inconsistent**	There is evidence for the stated association (inequalities, inverse, or mixed), but this is not found across all primary studies in the included systematic reviews.

A table explaining how the findings of the included systematic reviews were classified and their definitions.

The extracted mechanisms were collated in a table to further support the synthesis. These mechanisms were then mapped onto an adapted version of Levesque *et al*’s. patient-centred access to healthcare framework [35] (see the additional material **S5 Appendix**). The framework was adapted for this review to depict the process of vaccination, for more explanation, please refer to the protocol [25]. Mapping the mechanisms onto the framework will provide an understanding of the trickle-down effects of socioeconomic inequalities on vaccine uptake and ascertain which key stages of the vaccination process are impacted. To make this process easier, a table version has been created (**Table 2**).

**Table 2:** Patient-centred access to vaccination framework, table version.

	Mediators	Explanation	Mechanism	Reference
**A** **↓**	**Approachability**	“Correct, unbiased information provided about vaccines and vaccination.”		
	**Ability/likelihood to approach**	“Health literacy and beliefs and trust in the benefits of vaccines and vaccination.”		
**B** **↓**	**Acceptability**	“Integrity, outward presentation of vaccine manufacturers and vaccination provider.”		
	**Ability/likelihood to accept**	“Personal, social, and cultural attitudes towards vaccine and vaccination.”		
**C** **↓**	**Accessibility**	“Geographic location and opening times of vaccination provider.”		
	**Ability/likelihood to access**	“Perceived quality of vaccination provider. Transport to vaccination provider location.”		
**D** **↓**	**Affordability**	“Direct, indirect, and opportunity costs of vaccines and vaccination programmes.”		
	**Ability/likelihood to pay**	“Method of payment (insurance, taxation, out-of-pocket).”		
**E** **↓**	**Affects**	“Service satisfaction. Reducing the impact or occurrence of VPD.”		
	**Likelihood of positive affects**	“Protection against vaccine-preventable diseases. Positive experience.”		

A table version of the patient-centred access to vaccination framework presented in the additional material **S5 Appendix** [25]. The framework was created to map the potential mechanisms explaining the link between socioeconomic status and vaccination.

## Results

### Searching

**Fig 1:**
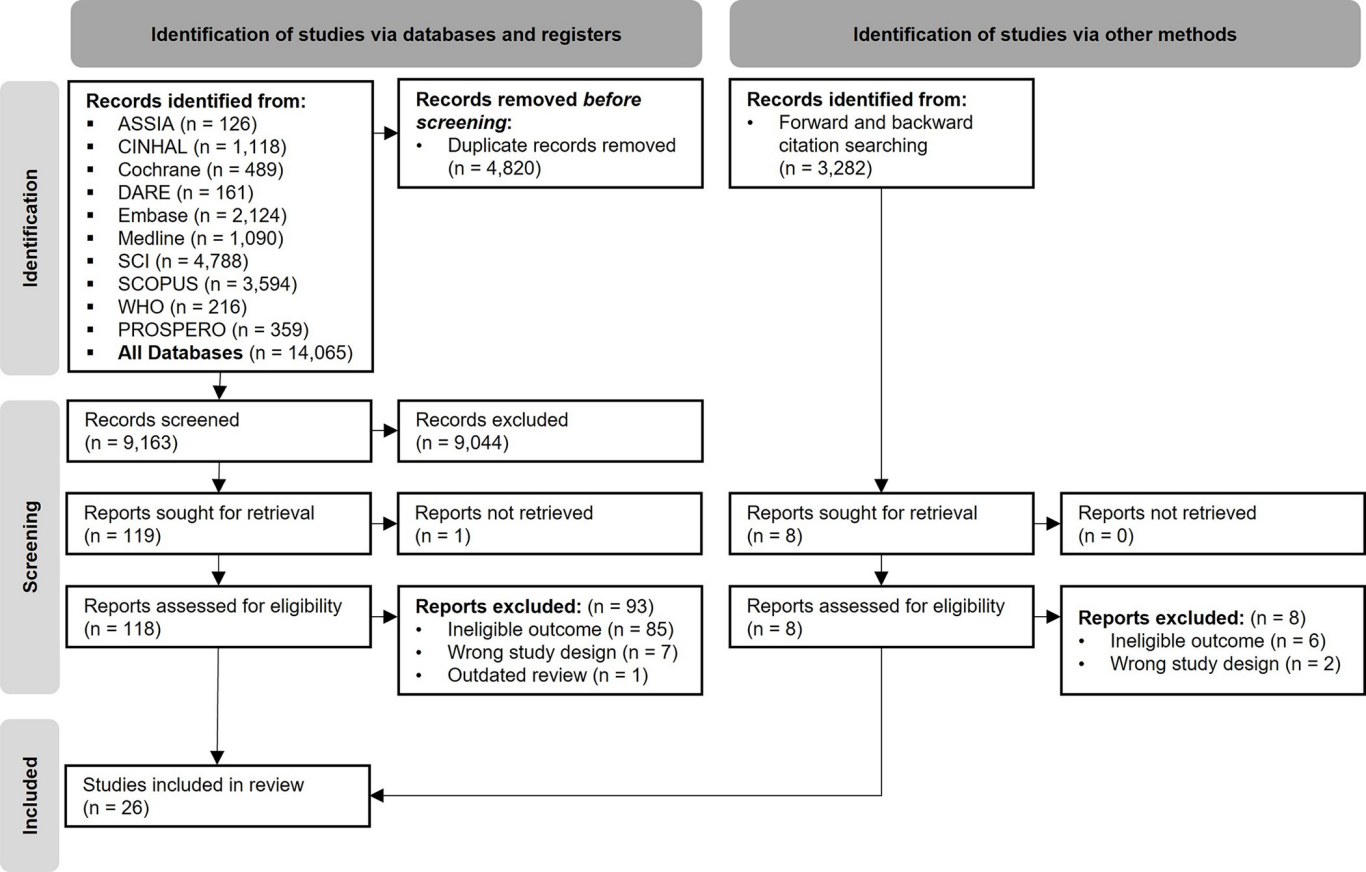
PRISMA-flow diagram. A completed PRISMA-flow diagram depicting the searching and screening process.

In total, 14,065 results were retrieved across the databases, and after deduplication, 9,163 results remained. After title and abstract screening 119 full-text reports were assessed for eligibility leaving a total of 26 systematic reviews meeting the inclusion criteria (**Fig 1**). Exclusion reasons for each of the 119 articles read at full-text stage are shown in the additional material, **S6 Appendix**. Forwards and backwards citation searching identified an additional 3,282 results, although after title and abstract screening (n = 3,282) and full text checking (n = 8) no further systematic reviews were eligible for inclusion. During this process we did not find any systematic reviews not published in English that were eligible for inclusion.

A total of 691 primary studies were included across all included reviews. Of the 691, 93 were included in more than one review, meaning there were 598 unique primary studies. The CCA was 13.46%, showing a moderate level of overlap of the primary studies.

### Quality appraisal

Of the 26 reviews, all were deemed of “critically low” quality. The verdict for each AMSTAR-2 question can be seen in the additional material, **S4 Appendix**. Some key areas in which the included reviews frequently scored poorly were as follows:

Question 2—not containing an explicit statement that the review methods were established prior to conducting the review (such as a protocol, or a study registration database) (n = 22).

Question 7—not including a full list of excluded primary studies and their reasoning (n = 26).

Question 10—not providing the funding details of each primary study (n = 24).

Question 13—not accounting for risk of bias in the interpretation/discussion of results (n = 21).

Please refer to Shea *et al*’s. AMSTAR-2 guidance [31] regarding the implications of scoring low on these four items. Indeed, failing to provide the funding details of included primary studies is a crucial oversight in the context of vaccination. The far-reaching repercussions of this failure were starkly evident in 1998 with the MMR vaccine [36], the effects of which still persist today.

### Summary of included studies

Of all the included reviews, a meta-analysis was conducted on 7 occasions [13, 17, 37–41], and a narrative approach was taken in the remaining 18 reviews [11, 12, 15, 18, 42–54]. One of the reviews that conducted a meta-analysis as its main method of synthesis, but the findings in relation to socioeconomic inequalities were only synthesised narratively [38]. The remaining study, by Ali *et al*., performed both a meta-analysis and narrative synthesis [55].

The included systematic reviews covered a range of contexts. Several countries and geographical groupings were included: n = 14 high-income countries [11–13, 15, 18, 38, 42, 44, 45, 47–49, 52, 56], n = 7 low/middle-income countries [37, 39, 41, 51, 54, 55], and n = 5 a mix of high/low/middle-income countries [17, 40, 46, 50, 53].

The included vaccines were as follows: childhood/adolescent routine schedule, in whole or in part (n = 10), HPV (n = 10), influenza and/or pneumococcal (n = 5), and all routine vaccinations (childhood and adult) (n = 1). Moreover, uptake was referred to, and measured in, a variety of different ways. Eight reviews did not define how they measured uptake [11, 38, 40, 41, 43, 47, 50, 55], although five of these explored influenza vaccination which has no set schedule [38, 40, 41, 47, 50]. One review reported vaccine initiation [52], and two reported vaccine schedule completion [51, 54]. The remaining 15 reviews measured both vaccine initiation and completion [12, 13, 15, 17, 18, 37, 39, 42, 44–46, 48, 49, 53, 56].

The populations across the reviews varied, due to the differing vaccines and their respective target groups. Amongst the publications that focused on childhood vaccines, n = 3 were of children under 2 years of age [37, 39, 53], n = 3 under 5 years [11, 43, 51], n = 1 under 7 years [56], n = 2 under 12 years [17, 18], and n = 1 unspecified [54]. For the reviews that examined HPV vaccination, n = 5 focused on females [12, 13, 44, 45, 49], n = 1 on males [52], and n = 4 on both females and males [15, 42, 46, 48]. Moreover, for the reviews that examined influenza vaccination, n = 2 explored adults aged 65 years and under [40, 50], while n = 4 reviews had no population restrictions [38, 41, 47, 55].

The findings relating to SES were reported in one of two ways; either according to each measure, or under a subheading of “socioeconomic status” incorporating all measures. Five reviews used one measure of SES (n = 1 area-level deprivation [42], n = 4 education [17, 39, 41, 46]); while 21 reviews utilised two, three, or four measures (SES, education, income/wealth, various area-level, occupation/employment). These measures were operationalised in vastly different ways, although it was common for reviews focusing on children or adolescents to refer only to maternal education, rather than a combined parental measure. A ‘Characteristics of Included Studies’ table can be seen in the additional material, **S7 Appendix**, which details the specific vaccines, geographical areas, populations, and measures of SES.

### Socioeconomic inequalities and vaccine uptake

The association between socioeconomic status and vaccination uptake were summarised in a matrix-style table, (**Table 3**). All 26 reviews reported an association between SES and vaccination uptake. Evidence for inequalities (advantaged SES, higher vaccination uptake, OR disadvantaged SES, lower vaccination uptake) were found on all but two occasions where only inverse associations were identified (n = 24). However, in over half of these reviews (n = 15) the overall conclusions were that of mixed findings as support for inverse associations were also identified. In total, 17 reviews found evidence for inverse associations: either lower vaccination uptake for advantaged SES (n = 6) higher uptake for disadvantaged SES groups (n = 7), or both (n = 4).

**Table 3:** Summary of findings.

	Inequalities	Inverse	Mixed
	Advantaged SES, higher vaccination uptake, AND/OR disadvantaged SES, lower vaccination uptake.	Disadvantaged SES, higher vaccination uptake (↓), AND/OR advantaged SES, lower vaccination uptake (↑), both (↕)	Evidence of inequalities and inverse associations.
**High-income countries**	**Gallagher [15]** (Narrative) (n = 14)^1^	**Dyda [38]** (Narrative) (n = 2)^2^ ↓	**Arat [11]** (Narrative) (n = 15)^1^ ↑
	**Fisher [13]** (Meta-analysis) (n = 19)^1^	**Mansfield [48]** (Narrative) (n = 5)^2^ ↕	**Bocquier [18]** (Narrative) (n = 34)^1^ ↑
			**Do [42]** (Narrative) (n = 11)^2^ ↓
			**Fernandez [12]** (Narrative) (n = 16)^1^ ↑
			**Galbraith [44]** (Narrative) (n = 4)^1^ ↓
			**Kessels [45]** (Narrative) (n = 11)^1^ ↓
			**Lucyk [47]** (Narrative) (n = 22)^1^ ↓
			**Murfin [49]** (Narrative) (n = 6)^1^ ↕
			**Schellenberg [56]** (Narrative) (n = 8)^1^ ↓
			**Shin [52]** (Narrative) (n = 14)^1^ ↕
**Low, middle-income countries**	**Desalew [37]** (Meta-analysis) (n = 28)^1^		**Ali [55]** (Meta-analysis/Narrative) (n = 87)^1^ ↑
	**Eshete [39]** (Meta-analysis) (n = 30)^2^		**Shenton [51]** (Scoping) (n = 125*)^1^ ↕
	**Galadima [43]** (Narrative) (n = 15)^2^		
	**Tilahun [54]** (Narrative) (n = 15)^1^		
	**Wang [41]** (Meta-analysis) (n = 25)^2^		
**High, middle, low-income countries**	**Forshaw [17]** (Meta-analysis) (n = 37)^1^		**Loke [46]** (Narrative) (n = 7)^2^ ↑
	**Okoli [40]** (Meta-analysis) (n = 20)^1^		**Nagata [50]** (Narrative) (n = 10)^1^ ↓
			**Tauil [53]** (Narrative) (n = 10)^2^ ↑
**Key:**			
Human Papillomavirus vaccination (HPV).	Influenza and/or Pneumococcal vaccination.	All routine vaccinations (childhood and adult).	Childhood/adolescent vaccination schedule.

A summary of the evidence exploring the association between socioeconomic status and vaccinations uptake, across all countries and vaccine. *n* = number of relevant primary studies.

^**1**^Inconsistent associations (There is evidence for the stated association (inequalities, inverse, or mixed), but this is not found across all primary studies in the included systematic review).

^**2**^Consistent associations (There is evidence for the stated association (inequalities, inverse, or mixed) across all primary studies in the included systematic review).

Disadvantaged SES, higher vaccination uptake (↓), AND/OR advantaged SES, lower vaccination uptake (↑), both (↕).

*125 primary studies were included in the scoping review, but only a percentage of relevant studies were provided, not an exact number.

Overall, the differing measures of SES employed by the reviews did not seem to explain their varying conclusions. Income, education, occupation/employment, and area-level deprivation were neither more, nor less, frequently cited as being associated with vaccination uptake. Additionally, mixed findings were equally prevalent across all measures; evidence for inequalities and/or inverse associations were not restricted to a specific aspect of SES. This result is also applicable across different vaccinations. Across the 26 reviews, there was no evidence to suggest that a particular vaccine, or group of vaccines, were more, or less, prone to inequalities in uptake by SES. Mixed results (evidence of inequalities and inverse associations) were equally common for all vaccinations.

### Country context

Overall, the association between SES and vaccination uptake was more complex among high-income countries than in low/middle-income countries. In high-income countries evidence for inverse associations and inequalities were both identified frequently.

### Low/Middle-income countries

Seven reviews focused on low/middle-income countries [37, 39, 41, 43, 51, 54, 55]. Five of these reviews explored the childhood/adolescent vaccination schedule [37, 39, 43, 51, 54]; two found evidence for consistent inequalities [39, 43], another two inconsistent inequalities [37, 54], and one had mixed findings [51]. In a review analysing influenza vaccination, a conclusion of consistent inequalities was made [41]. The seventh review, by Ali *et al*., explored the uptake for all routine vaccinations (childhood/adolescent and adulthood) and conducted both a narrative synthesis and meta-analysis [55]. The narrative synthesis showed consistent, mixed results, whereas the meta-analysis demonstrated consistent evidence for inequalities. Overall, in the context of low/middle-income countries, the findings largely suggest there are socioeconomic inequalities in vaccination uptake across the childhood/adolescent schedule, routine, and influenza vaccinations.

### High-income countries

The majority (n = 14) reviews focused exclusively on high-income countries [11–13, 15, 18, 38, 42, 44, 45, 47–49, 52, 56]; four of which analysed the childhood/adolescent vaccination schedule, finding mixed and inconsistent associations with SES in three [11, 18, 56], and one inconsistent evidence for inequalities [15]. A further eight reviews explored HPV vaccination [12, 13, 42, 44, 45, 48, 49, 52]; one found consistent evidence for inverse associations with SES [48], one inconsistent inequalities [13], and one consistent mixed [42]. The results of the remaining five reviews that explored HPV vaccination were inconsistent and mixed [12, 44, 45, 49, 52]. One review, analysing influenza and pneumococcal vaccination uptake, identified some consistent support for inverse associations with SES [38]. Analysing influenza vaccination, Lucyk *et al*. found inconsistent and mixed results [47]. Broadly, these results suggest that, among high-income countries, vaccination uptake does vary across different socioeconomic groups, but the results are often mixed and inconsistent. These findings appear to be applicable to all vaccines (the childhood/adolescent schedule, HPV, influenza, and pneumococcal) routinely given across high-income countries.

### High/middle/low-income countries

Five reviews explored a mixture of high, middle, and low-income country contexts [17, 40, 46, 50, 53]. Two reviews focused on the childhood/adolescent schedule [17, 53]; one identified inconsistent support for SES inequalities in uptake [17], whereas the other found consistently mixed associations [53]. Another review found consistent and mixed results for SES and HPV vaccination [46]. Okoli *et al*. and Nagata *et al*. analysed the uptake of influenza vaccination [40, 50]; the former identified inconsistent support for SES inequalities [40], whereas the latter showed inconsistent evidence for mixed associations [50]. Overall, reviews conducted in the context of high/middle/low-income countries identified mixed and inconsistent differences in vaccination uptake across different socioeconomic groups across all vaccines analysed (the childhood/adolescent schedule, HPV, influenza, and pneumococcal.

### Mechanisms

The majority (n = 16) of included reviews described potential mechanisms that could explain the differences in uptake according to SES. The following mechanisms were hypothesised had not been tested by the review authors: vaccine cost (n = 2) [18, 48]; access to transport (n = 3) [18, 37, 43]; time costs (n = 1) [18]; the extent of maternal control over household resources (n = 1) [43]; confidence (in vaccination in general, or in oneself to make decisions about uptake) (n = 6) [18, 37, 46, 48, 50, 56]; commitment to health-seeking behaviour (n = 3) [18, 43, 46]; vaccination knowledge (access to relevant information and/or ability to understand information) (n = 8) [12, 18, 37, 41, 43, 50, 56]; attitudes or beliefs about vaccination (n = 3) [43, 48, 56]; trust in healthcare or vaccination providers (n = 3) [46, 50, 56]; ease of access (based on the type of healthcare system) (n = 3) [17, 40, 55]; the vaccine delivery strategy (facility versus school-based) (n = 2) [15, 49]; funding of the vaccination programme (n = 2) [47, 53]. The identified mechanisms have been mapped on to the adapted patient-centred access to vaccination framework (**Table 4**).

**Table 4:** Extracted mechanisms mapped onto the patient-centred access to vaccination framework.

	Mediators	Explanation	Mechanism	Reference
**A** **↓**	**Approachability**	“Correct, unbiased information provided about vaccines and vaccination.”		
	**Ability/likelihood to approach**	“Health literacy and beliefs and trust in the benefits of vaccines and vaccination.”	Commitment to health-seeking behaviour.	[18, 43, 46]
			Vaccination knowledge (access to relevant information and/or ability to understand information).	[12, 18, 37, 41, 43, 50, 56]
**B** **↓**	**Acceptability**	“Integrity, outward presentation of vaccine manufacturers and vaccination provider.”		
	**Ability/likelihood to accept**	“Personal, social, and cultural attitudes towards vaccine and vaccination.”	Confidence (in vaccination in general, or in oneself to make decisions about uptake).	[18, 37, 46, 48, 50, 56]
			Extent of maternal control over household resources.	[43]
			Attitudes/beliefs about vaccination.	[43, 48, 56]
**C** **↓**	**Accessibility**	“Geographic location and opening times of vaccination provider.”	Vaccine delivery strategy (facility versus school-based).	[15, 49]
			Ease of access (based on the type of healthcare system).	[17, 40, 55]
	**Ability/likelihood to access**	“Perceived quality of vaccination provider. Transport to vaccination provider location.”	Access to transport.	[18, 37, 43]
			Trust in healthcare or vaccination provider.	[46, 50, 56]
**D** **↓**	**Affordability**	“Direct, indirect, and opportunity costs of vaccines and vaccination programmes.”	Funding of vaccination programme.	[47, 53]
	**Ability/likelihood to pay**	“Method of payment (insurance, taxation, out-of-pocket).”	Vaccine cost.	[18, 48]
			Time costs.	[18]
**E** **↓**	**Affects**	“Service satisfaction. Reducing the impact or occurrence of VPD.”		
	**Likelihood of positive affects**	“Protection against vaccine-preventable diseases. Positive experience.”		

Potential mechanisms explaining the link between socioeconomic status and vaccination uptake, as identified by the included reviews, mapped onto the patient-centred access to vaccination framework, presented in the additional material **S5 Appendix**, in tabular form [25].

## Discussion

This umbrella review had two aims; the first was to ascertain whether there are socioeconomic inequalities in vaccine uptake and summarize the contexts in which they exist. The second was to identify any mechanisms that could potentially explain these inequalities. Overall, the review illustrates that the literature surrounding this topic is complex, but there appears to be evidence for socioeconomic inequalities in vaccination uptake. In summary: (1) in the context of low-income countries, there appears to be consistent evidence for inequalities–lower vaccine uptake amongst disadvantaged SES groups, or higher vaccine uptake amongst advantaged SES groups; (2) for high-income or mixed (low/middle/high-income countries), the picture was more variable, with some evidence for inequalities and inverse associations (either low uptake for advantaged SES groups, or high uptake for disadvantaged SES groups); (3) mechanisms that may explain the association between SES and vaccination uptake mentioned in the majority (n = 16) reviews. The two most frequently cited mechanisms were vaccination knowledge (access to relevant information and/or ability to understand information) (n = 7) and confidence (in vaccination in general, or in oneself to make decisions about uptake) (n = 6). Moreover, the AMSTAR-2 checklist rated all 26 systematic reviews as “critically low” quality. Other umbrella reviews which have used this tool have also found similar results; the verdicts were all either low or critically low [57, 58]. We briefly explore the implications of this in the limitations. Given that we decided to include both randomised control trials and randomised studies of interventions, AMSTAR-2 was the most appropriate choice for our review [31].

To understand the first finding of the umbrella review—consistent evidence for inequalities in vaccination uptake in low/middle-income countries—it is important to appreciate the context. According to UNICEF, of the 25 million children who were under-vaccinated in 2021, more than 60% (15 million) reside in just 10 low/middle-income countries (India, Nigeria, Indonesia, Ethiopia, Philippines, Democratic Republic of the Congo, Brazil, Pakistan, Angola, and Myanmar) [59]. In the context of low uptake overall, inequalities may be more apparent. On the other hand, another explanation could be related to the role of education in low/middle-income countries. In their meta-analysis, Forshaw *et al*. found that the effect of higher maternal education on complete childhood vaccination was lower in Europe than in Asia or Africa [17]. Thus, the authors suggested that maternal education may play a more important role in lower-income countries than high-income countries.

However, in the context of high-income countries, higher levels of education can also be associated with lower uptake. In their systematic review of attitudes towards HPV vaccination in the United States, it was suggested by Mansfield *et al*. that “Parents’ educational attainment and vaccine beliefs may explain lower vaccination rates among high‐income families.” [48 p. 485]. Namely, that as level of education increases, there may be a greater commitment to health-seeking behaviour, which can either have a positive or negative affect on uptake [18, 43, 46]. This may help to understand the second finding of the umbrella review (in the context of high-income countries, the associations between vaccination uptake and SES were more variable than in low/middle-income countries). Thus, level of education is not necessarily more important in low/middle-income countries than in high-income countries, because lower uptake is also found to be associated with educationally advantaged groups within these high-income settings. In regard to higher uptake amongst disadvantaged SES groups, Mansfield *et al*. suggested that this may be the result of eligibility for government-funded healthcare assistance [48]. Also, influenza vaccination is funded by the government in Australia, where both a disadvantaged income and education were found to be associated with a greater odds of uptake, when compared to more advantaged groups [38]. There are several factors, therefore, that may contribute to the association between SES and vaccination uptake. To fully understand how these associations manifest, the country-context must be considered.

Despite many of the included reviews exploring multiple measures of SES, any discussions surrounding potential mechanisms were often understood by authors as being linked via level of education. Whilst this is interesting interpretation, the included systematic reviews fall short in explaining the link between different measures of SES and vaccination uptake. Nevertheless, we ascertained that SES affects several different stages of the vaccination process, as illustrated in **Table 4** where the mechanisms identified in the umbrella review were mapped on to the patient-centred access to vaccination framework. The framework helps to understand the considerations of both the service user and service provider. The two most frequently cited mechanisms were vaccination knowledge (access to relevant information and/or ability to understand information) (n = 7) and confidence (in vaccination in general, or in oneself to make decisions about uptake) (n = 6). In their global systematic review, Loke *et al*. identified mixed findings, suggesting that a “complex interplay may exist among education, vaccine concerns, and trust” [56 p. 581]. Knowledge and confidence are both referenced in the wider literature as having an impact on uptake. For instance, a systematic review investigating parental views of the HPV vaccine, Marshall *et al*. identified five themes in their analysis: (1) is prevention better than cure, (2) the fear of the unknown, (3) limited knowledge and understanding, (4) complex vaccination decisions, and (5) parental responsibility [60]. Although the wider literature is aware of these mechanisms, our umbrella review demonstrates how they can be linked to socioeconomic inequalities and which stages of the vaccination process they occur.

**Fig 2:**
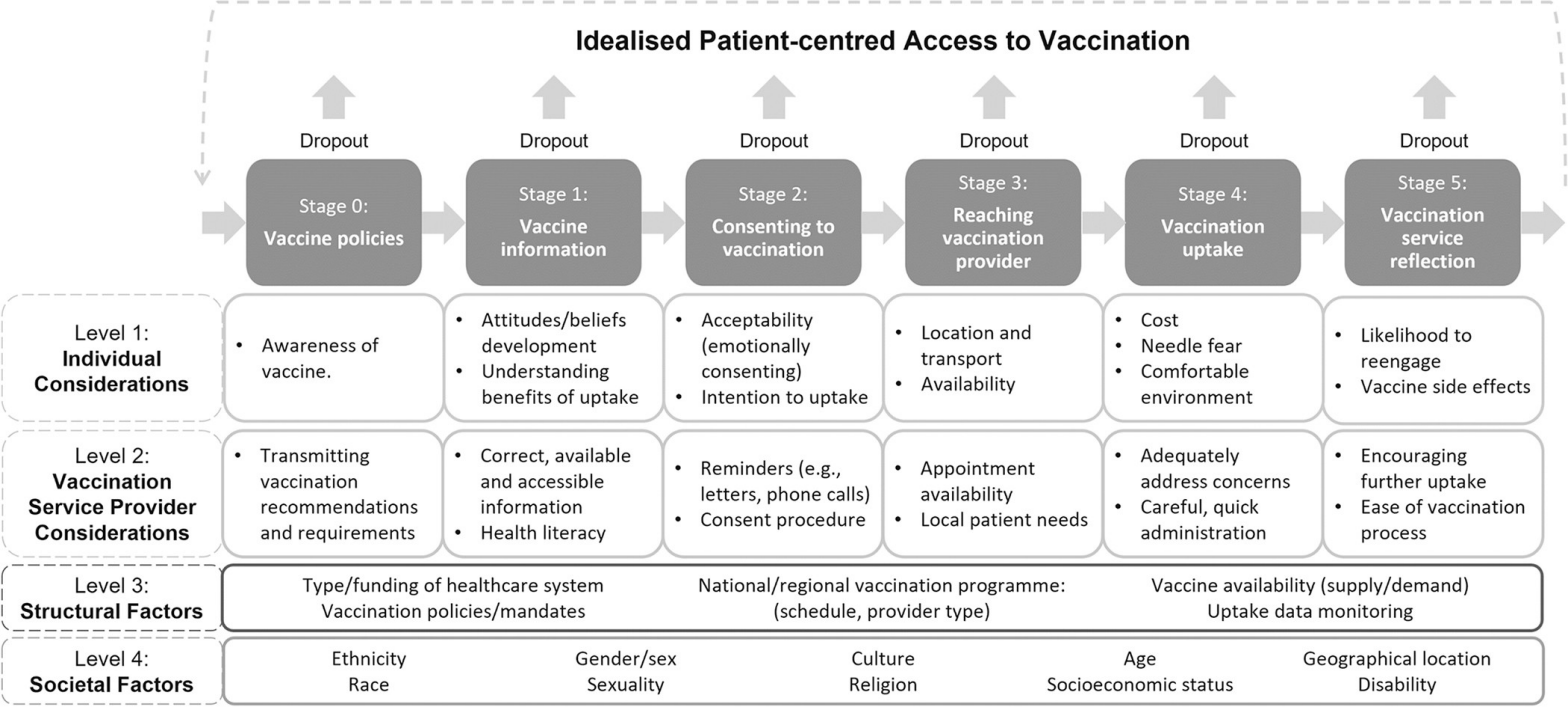
Framework conceptualising patient-centred access to vaccination, 2.0. An improved framework conceptualising patient-centred access to vaccination, informed by the findings of the umbrella review.

The patient-centred access to vaccination framework has been adapted to reflect the findings of the umbrella review (**Fig 2**). The updated version includes two more “levels”–“structural factors” (e.g., type/funding of healthcare system) and “societal factors” (e.g., socioeconomic status).

### Implications of the findings

This umbrella review has summarised and synthesised a large body of complex literature. It provides a concise description of the association between vaccination uptake and SES globally. The existing literature has acknowledged this complexity, particularly around the different directions of association between socioeconomic status and vaccine uptake [19]. However, what other literature fails to do is adequately portray the extent of inverse associations. This is especially relevant to high-income countries. By collating the evidence from several systematic reviews, a wider perspective reveals that higher uptake for disadvantaged SES groups, and lower uptake for advantaged SES, can occur. Before designing interventions to increase uptake, policy makers must be aware of the nature of the association between SES and specific vaccines within their population. Interventions must target all individuals with low uptake, which may include advantaged SES groups as well as disadvantaged SES groups.

Additionally, the exploration of the mechanisms linking SES and vaccination uptake raises questions about the legitimacy of linking consistently relating low uptake to education, as this neglects the structural barriers that are present. Our review highlights the need for more research into the legitimacy of these claims.

### Limitations

There are three main limitations to this umbrella review. Three minor amendments have been made to the methodology since the publication of the protocol [25]. Firstly, the study design inclusion criteria were broadened to allow for studies that analysed secondary data to be eligible. Many identified systematic reviews synthesised studies that analysed secondary data from national or regional vaccination registries. Excluding reviews that did would significantly reduce the number eligible. Secondly, the inclusion criteria were reframed using PECOS, in replacement of PICOS, as the former more accurately reflects what we aimed to explore. Thirdly, a pilot of the narrative synthesis indicated that an additional stage was required to adequately answer the primary research question. Namely, to ascertain whether certain countries demonstrated more evidence for differences in vaccination uptake according to SES. The results were organised by high-income, low/middle-income, or mixed, to address this concern.

Secondly, each level of synthesis (primary study to systematic review, systematic review to umbrella review) requires additional interpretation. Subsequently, there may be discrepancies in the conclusions made by the primary study authors and the systematic review authors. These potential discrepancies have then been collated for the purpose of this umbrella review. This issue is even more pertinent when taking into account that all included systematic reviews were rated “critically low” by the AMSTAR-2 checklist. However, it is important to note that AMSTAR-2 is a blunt instrument, with reviews undergoing an automatic downgrade if they do not satisfy one of the “critical domains” [32], and this does not reflect the quality of the primary studies included in these reviews.

Thirdly, there were some overlaps of primary studies, where they had been synthesised in more than one included systematic review. The CCA (13.46%) was reported, showing a moderate degree of overlap. Although the potential overlap was investigated, no action was taken to address the issue.

### Suggestions for future research

Future research on vaccine uptake must be more explicit in detailing their PICO criteria. At minimum, the vaccine should be stated, the number of doses (including the number required for full immunization), and the target age of administration–this is especially relevant when comparing multiple countries as routine schedules are likely to vary. Furthermore, it is important for authors conducting systematic reviews to give more careful consideration to the assessment tools used to appraise their work, such as AMSTAR-2. In doing so, they will have a greater awareness of the criterion which they should satisfy in order to be awarded a higher rating.

Moreover, the legitimacy of the mechanisms could be explored. This could be done through data analysis (if appropriate data are available), or via qualitative methods of inquiry. For instance, in socioeconomically deprived areas where vaccination uptake is low, the lived experience of these inequalities could be explored, both from the perspective of healthcare providers and service users.

## Conclusion

In conclusion, this umbrella review has highlighted the complexity of the potential association between vaccination uptake and SES. Although the quality of the included reviews is limited, there is some evidence to suggest that there are differences in vaccination uptake according to SES. Nevertheless, these associations do not consistently follow a clear gradient. Level of education was frequently mentioned by review authors as being the driving force behind SES differences in uptake, and the link to the identified mechanisms. Vaccination providers and policy makers must be intimately aware of how these differences manifest in their society to design effective interventions to increase uptake. These interventions should target any groups where uptake is low, even if this is amongst advantaged groups. Maximizing uptake subsequently increases herd immunity which widely beneficial for both those protected from disease and healthcare authorities. The recent pandemic has fuelled an alarming decrease in vaccination uptake globally, action needs to be taken to prevent further decline.

## Supporting information

S1 Appendix Completed PRISMA-E checklist. (DOCX)

S2 Appendix Detailed inclusion and exclusion criteria. (DOCX)

S3 Appendix Search strategy developed in Medline (Ovid). (DOCX)

S4 Appendix AMSTAR-2 quality appraisal questions and results for each included review. (DOCX)

S5 Appendix A framework depicting the process of patient-centred access to vaccination. (DOCX)

S6 Appendix Exclusion reasons for each of the identified, but ineligible, reviews. (DOCX)

S7 Appendix Characteristic of included studies. (DOCX)

## List of abbreviations

SES: SocioEconomic Status
PECOS: Population, Intervention, Comparison, Outcome, Study design
WHO: World Health Organization
AMSTAR-2: A MeaSurement Tool to Assess systematic Reviews
HPV: Human Papillomavirus
MMR: Measles-Mumps-Rubella
PRISMA-E: Preferred Reporting Items for Systematic Reviews and Meta-Analyses Equity
BCG: Bacillus Calmette-Guérin (Tuberculosis
DTP: containing–Diphtheria-Tetanus-Pertussis containing vaccine
Hib: Haemophilus Influenzae type B vaccine
PCV: Pneumococcal Conjugate Vaccine
IMD: Indices of Multiple Deprivation
DARE: Database of Abstracts of Reviews of Effects
SIA: Supplementary Immunisation Activities
CINAHL: Cumulated Index to Nursing and Allied Health Literature
SCI: Science Citation Index
ASSIA: Applied Social Sciences Index and Abstracts
CCA: Corrected Coverage Area
PICOS: Population, Intervention, Comparison, Outcome, Study design
