# Supplementary material for: Socioeconomic inequalities in vaccine uptake: A global umbrella review
Source: PLoS One. 2023 Dec 13;18(12):e0294688. doi: 10.1371/journal.pone.0294688 (PMC10718431; doi:10.1371/journal.pone.0294688)
Supplement: S2 Appendix — (DOCX) [file pone.0294688.s002.docx]

**S2 Appendix:** Detailed inclusion and exclusion criteria.

| Inclusion | Exclusion |
| --- | --- |
| Access to the full text. |  |
| Reviews published after 2011–present day. Any language (interpreters will be sourced if required). |  |
| **Inclusion:** Population   - All countries. - Normal/general populations. - Any demographic sub-population. | **Exclusion:** Population  Reviews which **focused** on:   - Occupational sub-populations (e.g., health care workers). - Clinically at-risk populations (e.g., diabetics and pregnant women). |
| **Inclusion:** Exposure – Advantaged SES  Reviews which focused on:   - Socioeconomic status, specifically, education, occupation, income, and area-level deprivation (any operationalization, e.g., years in education, or primary/secondary). | **Exclusion:** Exposure   - Any other measures of SES (e.g., receipt of state benefits, access to clean water, etc.). |
| **Inclusion:** Comparison – Disadvantaged SES   - Socioeconomic status, specifically, education, occupation, income, and area-level deprivation (any operationalization, e.g., years in education, or primary/secondary). | **Exclusions:** Comparison   - Any other measures of SES (e.g., receipt of state benefits, access to clean water, etc.). |
| **Inclusion:** Outcome  Reviews which **focused** on:   - Vaccine **uptake** (including either initiation and/or completion for multi-dose vaccines). - Schedule completion. - WHO-recommended routine vaccinations universally or worldwide (1). BCG (Tuberculosis), Hepatitis B, Polio, DTP-containing vaccine (Diphtheria, Tetanus and Pertussis), Haemophilus influenzae type b, Pneumococcal (conjugate), Rotavirus, Measles, Rubella, and HPV (Human papillomavirus). - Influenza and COVID-19 vaccinations, to account for reviews published in response to the 2019 Coronavirus pandemic. - Single-antigen or combined vaccines (Despite Mumps not being a universally recommended vaccine it was eligible for inclusion if explored as part of the combined MMR vaccine). | **Exclusion:** Outcome  Reviews which **focused** on:   - Interventions to improve vaccine uptake. - Vaccine uptake targets or estimation models. - Timeliness, supplementary immunisation activities (SIAs) or missed opportunities. - WHO vaccine recommendations for certain regions (Japanese Encephalitis, Yellow Fever, Tick-Borne Encephalitis) (1). - WHO vaccine recommendations for some high-risk populations (Typhoid, Cholera, Meningococcal, Hepatitis A, Rabies, and Dengue) (1). - WHO vaccine recommendations for immunisation programs with certain characteristics (Mumps and Varicella) (1). |
| **Inclusion:** Study Design   - Must be a systematic review, as defined by the DARE criteria (2). - Must synthesise primary empirical studies, or those which perform a secondary analysis on vaccine uptake monitoring data. | **Exclusion:** Study Design   - Studies which state they are reviews but do not meet four or more of the DARE criteria (2) or are a primary study or conference paper. - Mixed reviews where the relevant data could not be separated from the irrelevant, or erroneous, information. |
| (1) World Health Organization. Table 1: Summary of WHO Position Papers - Recommendations for Routine Immunization. 2021. https://cdn.who.int/media/docs/default-source/immunization/immunization_schedules/table_1_feb_2023_english.pdf?sfvrsn=c7de0e97_11&download=true. Accessed 27 Jun 2023.  (2) Database of Abstracts of Reviews of Effects (DARE): Quality Assessed Reviews. https://www.ncbi.nlm.nih.gov/books/NBK285222/. Accessed 27 June 2023. | |
