# Supplementary material for: Socioeconomic inequalities in vaccine uptake: A global umbrella review
Source: PLoS One. 2023 Dec 13;18(12):e0294688. doi: 10.1371/journal.pone.0294688 (PMC10718431; doi:10.1371/journal.pone.0294688)
Supplement: S3 Appendix — (DOCX) [file pone.0294688.s003.docx]

**S3 Appendix:** Search strategy developed in Medline (Ovid).

**[Exposure]**

Title, Abstract, Key words=

socioeconomic or socio-economic or sociodemographic or sep or ses or class or

education or lifelong learning or life-long learning or human capital or school* or literacy or academic achievement or

employ* or unemploy* or occupation* or job* or work or career* or vocation or economic activity or labour market activity or isco or

income or wealth or wage* or salar* or earning* or low-income or money or

(inequit* or inequalit* or unequal or equal* or equit* or depriv* or poverty or impoverished or disadvantage* or gradient or gap* or disparit* or difference*) adj3 economic

**[Outcome]**

AND

Title, Abstract, Key words=

vaccine* or immunize or immunise or injection* or jab* or inoculate or

(tb or tuberculosis or Hep B or Hepatitis B or diphtheria or tetanus or pertussis or whooping cough or hib or haemophilus or haemophilus influenzae type b or poliovirus or polio or poliomyelitis or pneumococcal or pneumococcus or rotavirus or measles or rubella or human papillomavirus or wart virus or influenza or flu or COVID-19 or COVID 19 or COVID19 or coronavirus or SARS-CoV-2 or SARS Cov 2 or severe acute respiratory syndrome) adj3 vaccine* or

(BCG or HepB or IPV or DTP-containing or DTPCV or Td or DT or DTaP or Tdap or PCV or RV or MMR or MR or HPV or IIV or LAIV) adj3 vaccine*

**[Outcome]**

AND

Title, Abstract, Key words=

vaccination or immunization or immunisation or inoculation or uptake or coverage or rate* or accept* or hesitan* or access

**[Study Design]**

AND

Title, Abstract, Key words=

systematic review* or systematic literature review or systematic overview or meta analys* or metaanalys* or review

**[Filters]**

Publication date 2011-present
