# Supplementary figures and images for: Socioeconomic inequalities in vaccine uptake: A global umbrella review
Source: PLoS One. 2023 Dec 13;18(12):e0294688. doi: 10.1371/journal.pone.0294688 (PMC10718431; doi:10.1371/journal.pone.0294688)

**S5 Appendix:** A framework depicting the process of patient-centred access to vaccination.

**
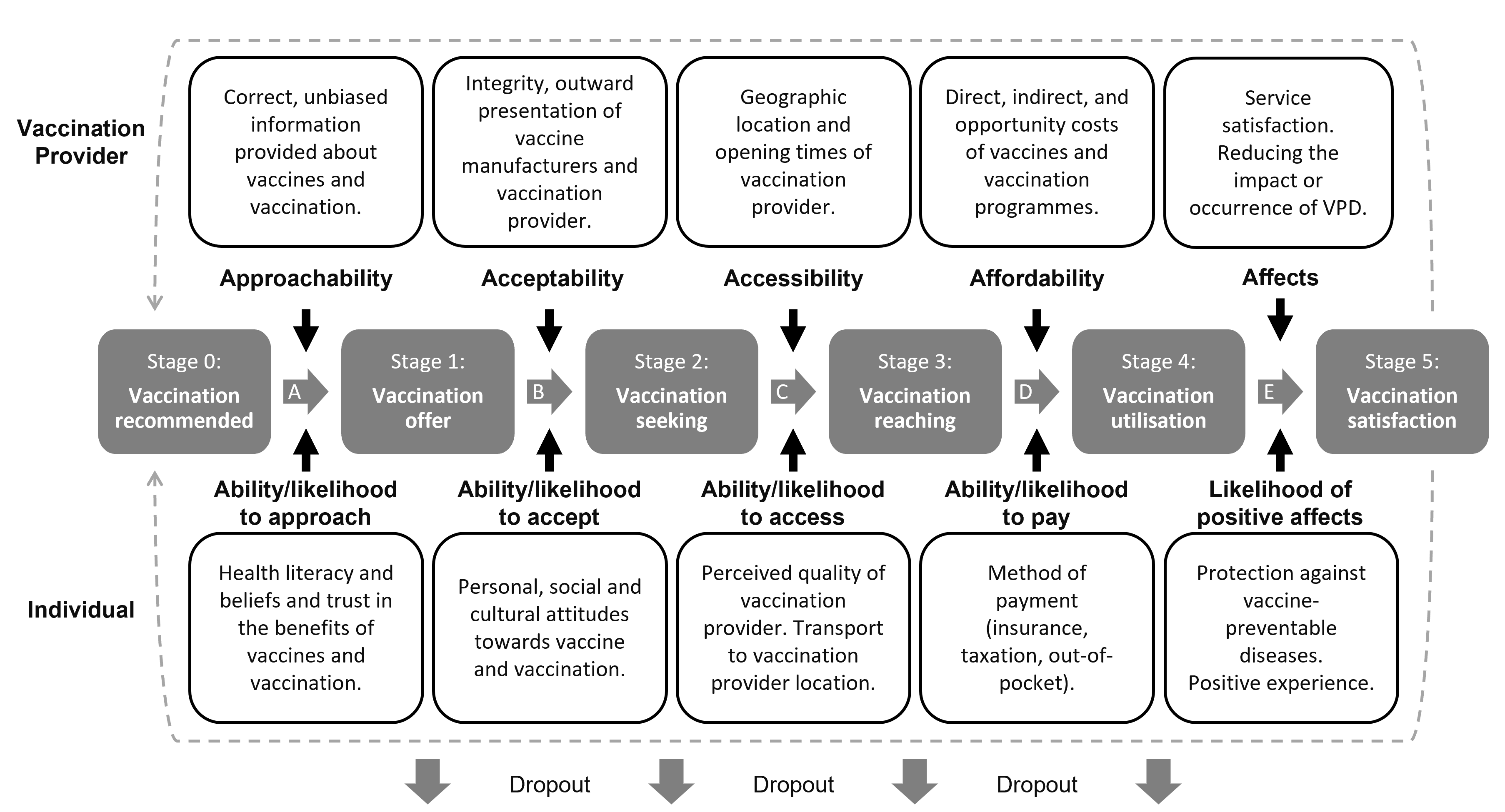
**

Supplement: S5 Appendix — (DOCX) [file pone.0294688.s005.docx]
