# Supplementary material for: Socioeconomic inequalities in vaccine uptake: A global umbrella review
Source: PLoS One. 2023 Dec 13;18(12):e0294688. doi: 10.1371/journal.pone.0294688 (PMC10718431; doi:10.1371/journal.pone.0294688)
Supplement: S6 Appendix — (DOCX) [file pone.0294688.s006.docx]

**S6 Appendix:** Exclusion reasons for each of the identified, but ineligible, reviews.

| **Title** | **Year** | **Authors** | **Notes** |  |
| --- | --- | --- | --- | --- |
| The Factors That Promote Vaccine Hesitancy, Rejection, or Delay in Parents | 2020 | Majid Umair and Ahmad Mobeen | RAYYAN-EXCLUSION-REASONS: focus on attitudes/perceptions | Wrong outcome |
| Human Papillomavirus Infection and Vaccination | 2016 | Valentino, Katie and Poronsky, Cathlin B | RAYYAN-EXCLUSION-REASONS: does not report socioeconomic inequalities in vaccine uptake | Wrong outcome |
| Public attitudes and influencing factors toward COVID-19 vaccination for adolescents/children: a scoping review | 2022 | Liu, Y and Ma, Q and Liu, H and Guo, Z | RAYYAN-EXCLUSION-REASONS: focus on attitudes/perceptions | Wrong outcome |
| Factors that influence parents' and informal caregivers' views and practices regarding routine childhood vaccination: a qualitative evidence synthesis | 2021 | Cooper, S and Schmidt, B-M and Sambala, EZ and Swartz, A and Colvin, CJ and Leon, N and Wiysonge, CS | RAYYAN-EXCLUSION-REASONS: focus on attitudes/perceptions | Wrong outcome |
| Barriers and facilitators to HPV vaccination of young women in high-income countries: A qualitative systematic review and evidence synthesis | 2014 | Ferrer, Harriet Batista and Trotter, Caroline and Hickman, Matthew and Audrey, Suzanne | RAYYAN-EXCLUSION-REASONS: does not report socioeconomic inequalities in vaccine uptake | Wrong outcome |
| "Hpv? Never heard of it!": a systematic review of girls' and parents' information needs, views and preferences about human papillomavirus vaccination | 2013 | Hendry, Maggie and Lewis, Ruth and Clements, Alison and Damery, Sarah and Wilkinson, Clare | RAYYAN-EXCLUSION-REASONS: does not report socioeconomic inequalities in vaccine uptake | Wrong outcome |
| Defining the determinants of vaccine uptake and undervaccination in migrant populations in Europe to improve routine and COVID-19 vaccine uptake: a systematic review | 2022 | Crawshaw A.F. and Farah Y. and Deal A. and Rustage K. and Hayward S.E. and Carter J. and Knights F. and Goldsmith L.P. and Campos-Matos I. and Wurie F. and Majeed A. and Bedford H. and Forster A.S. and Hargreaves S. | RAYYAN-EXCLUSION-REASONS: does not report socioeconomic inequalities in vaccine uptake | Wrong outcome |
| Factors affecting poor measles vaccination coverage in sub-Saharan Africa with a special focus on Nigeria: a narrative review | 2022 | Majekodunmi O.B. and Oladele E.A. and Greenwood B. | RAYYAN-EXCLUSION-REASONS: Mixed review: relevant information cannot be separated from the irrelevant | Wrong outcome |
| Prevalence and Determinants of COVID-19 Vaccine Hesitancy Among the Ethiopian Population: A Systematic Review | 2022 | Yehualashet D.E. and Seboka B.T. and Tesfa G.A. and Mamo T.T. and Yawo M.N. and Hailegebreal S. | RAYYAN-EXCLUSION-REASONS: focus on attitudes/perceptions | Wrong outcome |
| Determinants of COVID-19 vaccine acceptance in Ethiopia: A systematic review and meta-analysis | 2022 | Mose A. and Wasie A. and Shitu S. and Haile K. and Timerga A. and Melis T. and Sahle T. and Zewdie A. | RAYYAN-EXCLUSION-REASONS: focus on attitudes/perceptions | Wrong outcome |
| COVID-19 Vaccine Acceptance among Low-and Lower-Middle-Income Countries: A Rapid Systematic Review and Meta-Analysis | 2022 | Patwary M.M. and Alam M.A. and Bardhan M. and Disha A.S. and Haque M.Z. and Billah S.M. and Kabir M.P. and Browning M.H.E.M. and Rahman M.M. and Parsa A.D. and Kabir R. | RAYYAN-EXCLUSION-REASONS: focus on attitudes/perceptions | Wrong outcome |
| Acceptance of COVID-19 Vaccine and Its Associated Factors Among Ethiopian Population: A Systematic Review | 2022 | Bayou F.D. and Amare S.N. | RAYYAN-EXCLUSION-REASONS: focus on attitudes/perceptions | Wrong outcome |
| Impact of COVID-19 pandemic on routine vaccination coverage of children and adolescents: A systematic review | 2022 | SeyedAlinaghi S. and Karimi A. and Mojdeganlou H. and Alilou S. and Mirghaderi S.P. and Noori T. and Shamsabadi A. and Dadras O. and Vahedi F. and Mohammadi P. and Shojaei A. and Mahdiabadi S. and Janfaza N. and Keshavarzpoor Lonbar A. and Mehraeen E. and Sabatier J.-M. | RAYYAN-EXCLUSION-REASONS: does not report socioeconomic inequalities in vaccine uptake | Wrong outcome |
| Improving the Acceptability of Human Papillomavirus Vaccines Among Men Who Have Sex With Men According to the Associated Factors: A Systematic Review and Meta-analysis | 2021 | Zhao Y. and Xin X. and Deng H. and Xu J. and Weng W. and Zhang M. and Li J. and Gao Y. and Huang X. and Liu C. | RAYYAN-EXCLUSION-REASONS: focus on attitudes/perceptions | Wrong outcome |
| Acceptance of COVID-19 vaccination and correlated variables among global populations: A systematic review and meta-analysis | 2021 | Nindrea R.D. and Usman E. and Katar Y. and Sari N.P. | RAYYAN-EXCLUSION-REASONS: focus on attitudes/perceptions | Wrong outcome |
| Factors associated with the hpv vaccination among korean americans and koreans: A systematic review | 2022 | Jo S. and Han S.-Y. and Walters C.A. | RAYYAN-EXCLUSION-REASONS: does not report socioeconomic inequalities in vaccine uptake | Wrong outcome |
| Progress and barriers towards maternal and neonatal tetanus elimination in the remaining 12 countries: a systematic review | 2021 | Yusuf N. and Raza A.A. and Chang-Blanc D. and Ahmed B. and Hailegebriel T. and Luce R.R. and Tanifum P. and Masresha B. and Faton M. and Omer M.D. and Farrukh S. and Aung K.D. and Scobie H.M. and Tohme R.A. | RAYYAN-EXCLUSION-REASONS: does not report socioeconomic inequalities in vaccine uptake | Wrong outcome |
| Global COVID-19 Vaccine Acceptance: A Systematic Review of Associated Social and Behavioral Factors | 2022 | Shakeel C.S. and Mujeeb A.A. and Mirza M.S. and Chaudhry B. and Khan S.J. | RAYYAN-EXCLUSION-REASONS: focus on attitudes/perceptions | Wrong outcome |
| HPV vaccine: uptake and understanding among global Indigenous communities - a qualitative systematic review | 2021 | Poirier B. and Sethi S. and Garvey G. and Hedges J. and Canfell K. and Smith M. and Ju X. and Jamieson L. | RAYYAN-EXCLUSION-REASONS: focus on attitudes/perceptions | Wrong outcome |
| Vaccination uptake amongst older adults from minority ethnic backgrounds: A systematic review | 2021 | Bhanu C. and Gopal D.P. and Walters K. and Chaudhry U.A.R. | RAYYAN-EXCLUSION-REASONS: does not report socioeconomic inequalities in vaccine uptake | Wrong outcome |
| Hepatitis B vaccination coverage in Germany: systematic review | 2021 | Steffen G. and Sperle I. and Harder T. and Sarma N. and Beermann S. and Thamm R. and Bremer V. and Zimmermann R. and Dudareva S. | RAYYAN-EXCLUSION-REASONS: does not report socioeconomic inequalities in vaccine uptake | Wrong outcome |
| Disruptions to routine childhood vaccinations in low- and middle-income countries during the COVID-19 pandemic: A systematic review. | 2022 | Cardoso Pinto, Alexandra M and Ranasinghe, Lasith and Dodd, Peter J and Budhathoki, Shyam Sundar and Seddon, James A and Whittaker, Elizabeth | RAYYAN-EXCLUSION-REASONS: does not report socioeconomic inequalities in vaccine uptake | Wrong outcome |
| Covid-19 Vaccine Acceptance and Determinant Factors among General Public in East Africa: A Systematic Review and Meta-Analysis. | 2022 | Alemayehu, Astawus and Demissie, Abebaw and Yusuf, Mohammed and Gemechu Lencha, Abebe and Oljira, Lemessa | RAYYAN-EXCLUSION-REASONS: focus on attitudes/perceptions | Wrong outcome |
| Ethnic/racial minorities' and migrants' access to COVID-19 vaccines: A systematic review of barriers and facilitators. | 2022 | Abba-Aji, Mohammed and Stuckler, David and Galea, Sandro and McKee, Martin | RAYYAN-EXCLUSION-REASONS: does not report socioeconomic inequalities in vaccine uptake | Wrong outcome |
| Attitudes, acceptance and hesitancy among the general population worldwide to receive the COVID-19 vaccines and their contributing factors: A systematic review. | 2021 | Cascini, Fidelia and Pantovic, Ana and Al-Ajlouni, Yazan and Failla, Giovanna and Ricciardi, Walter | RAYYAN-EXCLUSION-REASONS: focus on attitudes/perceptions | Wrong outcome |
| Human papillomavirus vaccination uptake in low-and middle-income countries: a meta-analysis. | 2021 | Dorji, Thinley and Nopsopon, Tanawin and Tamang, Saran Tenzin and Pongpirul, Krit | RAYYAN-EXCLUSION-REASONS: does not report socioeconomic inequalities in vaccine uptake | Wrong outcome |
| The impact of the COVID-19 pandemic on immunization campaigns and programs: A systematic review | 2021 | Lassi Z.S. and Naseem R. and Salam R.A. and Siddiqui F. and Das J.K. | RAYYAN-EXCLUSION-REASONS: does not report socioeconomic inequalities in vaccine uptake | Wrong outcome |
| Vaccination against COVID-19: A systematic review and meta-analysis of acceptability and its predictors | 2021 | Wang Q. and Yang L. and Jin H. and Lin L. | RAYYAN-EXCLUSION-REASONS: focus on attitudes/perceptions | Wrong outcome |
| A scoping review to find out worldwide covid-19 vaccine hesitancy and its underlying determinants | 2021 | Biswas M.R. and Alzubaidi M.S. and Shah U. and Abd-Alrazaq A.A. and Shah Z. | RAYYAN-EXCLUSION-REASONS: does not report socioeconomic inequalities in vaccine uptake | Wrong outcome |
| A rapid systematic review of factors influencing covid-19 vaccination uptake in minority ethnic groups in the uk | 2021 | Kamal A. and Hodson A. and Pearce J.M. | RAYYAN-EXCLUSION-REASONS: does not report socioeconomic inequalities in vaccine uptake | Wrong outcome |
| [Human papillomavirus vaccine receptivity: a systematic reviewreceptividad con respecto a la vacuna contra el virus del papiloma humano: revision sistematica]. | 2019 | da Silva, Lidia Ester Lopes and de Oliveira, Maria Liz Cunha and Galato, Dayani | RAYYAN-EXCLUSION-REASONS: does not report socioeconomic inequalities in vaccine uptake | Wrong outcome |
| COVID-19 vaccine hesitancy in Africa: a scoping review. | 2022 | Ackah, Betty B B and Woo, Michael and Stallwood, Lisa and Fazal, Zahra A and Okpani, Arnold and Ukah, Ugochinyere Vivian and Adu, Prince A | RAYYAN-EXCLUSION-REASONS: focus on attitudes/perceptions | Wrong outcome |
| What is the state-of-the-art in clinical trials on vaccine hesitancy 2015-2020? | 2021 | Pires C. | RAYYAN-EXCLUSION-REASONS: focus on attitudes/perceptions | Wrong outcome |
| Vaccine attitudes among young adults in Asia: a systematic review | 2021 | Wang L. and Liang Y. and Zhang X. and Yang J. | RAYYAN-EXCLUSION-REASONS: Mixed review: relevant information cannot be separated from the irrelevant | Wrong outcome |
| Evaluation of the Acceptance Rate of Covid-19 Vaccine and its Associated Factors: A Systematic Review and Meta-analysis. | 2022 | Kazeminia, Mohsen and Afshar, Zeinab Mohseni and Rajati, Mojgan and Saeedi, Anahita and Rajati, Fatemeh | RAYYAN-EXCLUSION-REASONS: focus on attitudes/perceptions | Wrong outcome |
| Mmr vaccine attitude and uptake research in the united kingdom: A critical review | 2021 | Torracinta L. and Tanner R. and Vanderslott S. | RAYYAN-EXCLUSION-REASONS: not a systematic review, as defined by DARE | Wrong study design |
| COVID-19 vaccination hesitancy in Hispanics and African-Americans: A review and recommendations for practice. | 2021 | Khubchandani, Jagdish and Macias, Yilda | RAYYAN-EXCLUSION-REASONS: focus on hesitancy | Wrong outcome |
| Barriers and facilitators to HPV vaccine uptake among US rural populations: a scoping review | 2020 | Peterson C.E. and Silva A. and Holt H.K. and Balanean A. and Goben A.H. and Dykens J.A. | RAYYAN-EXCLUSION-REASONS: does not report the correct socioeconomic inequalities | Wrong outcome |
| Predictors of COVID-19 Vaccine Acceptance, Intention, and Hesitancy: A Scoping Review. | 2021 | Joshi, Ashish and Kaur, Mahima and Kaur, Ritika and Grover, Ashoo and Nash, Denis and El-Mohandes, Ayman | RAYYAN-EXCLUSION-REASONS: focus on hesitancy | Wrong outcome |
| Barriers to childhood immunization in sub-Saharan Africa: A systematic review | 2020 | Bangura J.B. and Xiao S. and Qiu D. and Ouyang F. and Chen L. | RAYYAN-EXCLUSION-REASONS: Mixed review: relevant information cannot be separated from the irrelevant | Wrong outcome |
| Vaccine acceptability, uptake and completion amongst men who have sex with men: A systematic review, meta-analysis and theoretical framework. | 2021 | Nadarzynski, Tom and Frost, Miles and Miller, Danny and Wheldon, Christopher W and Wiernik, Brenton M and Zou, Huachun and Richardson, Daniel and Marlow, Laura A V and Smith, Helen and Jones, Christina J and Llewellyn, Carrie | RAYYAN-EXCLUSION-REASONS: focus on attitudes/perceptions | Wrong outcome |
| Analysis of community-based studies related with knowledge, awareness, attitude, and behaviors towards hpv and hpv vaccine published in turkey: A systematic review | 2020 | Ozdemir S. and Akkaya R. and Karasahin K.E. | RAYYAN-EXCLUSION-REASONS: focus on attitudes/perceptions | Wrong outcome |
| Barriers to vaccination in Latin America: A systematic literature review | 2020 | Guzman-Holst A. and DeAntonio R. and Prado-Cohrs D. and Juliao P. | RAYYAN-EXCLUSION-REASONS: focus on attitudes/perceptions | Wrong outcome |
| Vaccine-preventable diseases and immunisation coverage among migrants and non-migrants worldwide: A scoping review of published literature, 2006 to 2016. | 2019 | Charania, Nadia A and Gaze, Nina and Kung, Janice Y and Brooks, Stephanie | RAYYAN-EXCLUSION-REASONS: does not report the correct socioeconomic inequalities | Wrong outcome |
| Determinants of Seasonal Influenza Vaccine Uptake Among the Elderly in the United States: A Systematic Review and Meta-Analysis | 2019 | Okoli G.N. and Abou-Setta A.M. and Neilson C.J. and Chit A. and Thommes E. and Mahmud S.M. | RAYYAN-EXCLUSION-REASONS: Old review, updated version included | Outdated review |
| Acceptance and uptake of influenza vaccines in Asia: A systematic review | 2019 | Sheldenkar A. and Lim F. and Yung C.F. and Lwin M.O. | RAYYAN-EXCLUSION-REASONS: does not report socioeconomic inequalities in vaccine uptake | Wrong outcome |
| Measles Status-Barriers to Vaccination and Strategies for Overcoming Them. | 2018 | Storr, Constanze and Sanftenberg, Linda and Schelling, Joerg and Heininger, Ulrich and Schneider, Antonius | RAYYAN-EXCLUSION-REASONS: not a systematic review, as defined by DARE | Wrong study design |
| Ensuring childhood vaccination among slums dwellers under the National Immunization Program in India - Challenges and opportunities. | 2018 | Singh, Sanjeev and Sahu, Damodar and Agrawal, Ashish and Vashi, Meeta Dhaval | RAYYAN-EXCLUSION-REASONS: not a systematic review, as defined by DARE | Wrong study design |
| Health literacy and vaccination: A systematic review. | 2018 | Lorini, Chiara and Santomauro, Francesca and Donzellini, Martina and Capecchi, Leonardo and Bechini, Angela and Boccalini, Sara and Bonanni, Paolo and Bonaccorsi, Guglielmo | RAYYAN-EXCLUSION-REASONS: wrong outcome | Wrong outcome |
| Facilitators and barriers for use of rotavirus vaccine amongst various stakeholders and its implications for Indian context-A systematic review | 2018 | Apte A. and Roy S. and Bavdekar A. and Juvekar S. and Hirve S. | RAYYAN-EXCLUSION-REASONS: focus on intention to vaccinate | Wrong outcome |
| Knowledge, awareness and acceptability of anti-hpv vaccine in the arab states of the middle east and north africa region: A systematic review | 2018 | Gamaoun R. | RAYYAN-EXCLUSION-REASONS: focus on attitudes/perceptions | Wrong outcome |
| Coverage and determinants of childhood immunization in Nigeria: A systematic review and meta-analysis. | 2017 | Adeloye, Davies and Jacobs, Wura and Amuta, Ann O and Ogundipe, Oluwatomisin and Mosaku, Oluwaseun and Gadanya, Muktar A and Oni, Gbolahan | RAYYAN-EXCLUSION-REASONS: wrong outcome | Wrong outcome |
| Human Papillomavirus Vaccination Uptake in Canada: A Systematic Review and Meta-analysis. | 2017 | Bird, Yelena and Obidiya, Olatunji and Mahmood, Razi and Nwankwo, Chijioke and Moraros, John | RAYYAN-EXCLUSION-REASONS: does not report the correct socioeconomic inequalities | Wrong outcome |
| Determinants of European parents' decision on the vaccination of their children against measles, mumps and rubella: A systematic review and meta-analysis. | 2016 | Tabacchi, Garden and Costantino, Claudio and Napoli, Giuseppe and Marchese, Valentina and Cracchiolo, Manuela and Casuccio, Alessandra and Vitale, Francesco and The Esculapio Working Group | RAYYAN-EXCLUSION-REASONS: Mixed review: relevant information cannot be separated from the irrelevant | Wrong outcome |
| Immunisation coverage in rural-urban migrant children in low and middle-income countries (lmics): a systematic review and meta-analysis. | 2016 | Awoh, Abiyemi Benita and Plugge, Emma | RAYYAN-EXCLUSION-REASONS: does not report the correct socioeconomic inequalities | Wrong outcome |
| Gender Determinants of Vaccination Status in Children: Evidence from a Meta-Ethnographic Systematic Review. | 2015 | Merten, Sonja and Martin Hilber, Adriane and Biaggi, Christina and Secula, Florence and Bosch-Capblanch, Xavier and Namgyal, Pem and Hombach, Joachim | RAYYAN-EXCLUSION-REASONS: does not report vaccine uptake | Wrong outcome |
| Antimeningococcal and antipneumococcal vaccination determinants: a European systematic literature review. | 2015 | Malerba, Valentina and Costantino, Claudio and Napoli, Giuseppe and Marchese, Valentina and Casuccio, Alessandra and Tabacchi, Garden and Vitale, Francesco and ESCULAPIOWorking Group and Amicizia D, Bechini A, Boccalini S, Bonanni P, Coppola R, Fortunato F, Gasparini R, Levi M, Martinelli D, Panatto D, Pellizzari B, Prato R | RAYYAN-EXCLUSION-REASONS: does not report the correct socioeconomic inequalities | Wrong outcome |
| HPV vaccine acceptability in Africa: a systematic review. | 2014 | Cunningham, Melissa S and Davison, Colleen and Aronson, Kristan J | RAYYAN-EXCLUSION-REASONS: focus on attitudes/perceptions | Wrong outcome |
| Facilitators and barriers to adult vaccination in south east asia and Latin America | 2017 | Davis B.M. and Black D. | RAYYAN-EXCLUSION-REASONS: conference abstract | Wrong study design |
| Reducing social inequalities in childhood vaccination uptake | 2017 | Uhomoibhi C. and Bedford H. and Pearce A. | RAYYAN-EXCLUSION-REASONS: wrong outcome | Wrong outcome |
| Immunization, urbanization and slums - a systematic review of factors and interventions | 2017 | Crocker-Buque T. and Mindra G. and Duncan R. and Mounier-Jack S. | RAYYAN-EXCLUSION-REASONS: Mixed reviews: relevant information cannot be separated from the irrelevant | Wrong outcome |
| Vaccinations in migrants and refugees: a challenge for European health systems. A systematic review of current scientific evidence | 2017 | Mipatrini D. and Stefanelli P. and Severoni S. and Rezza G. | RAYYAN-EXCLUSION-REASONS: does not report socioeconomic inequalities in vaccine uptake | Wrong outcome |
| HPV vaccine acceptability among men: a systematic review and meta-analysis. | 2013 | Newman, Peter A and Logie, Carmen H and Doukas, Nick and Asakura, Kenta | RAYYAN-EXCLUSION-REASONS: focus on attitudes/perceptions | Wrong outcome |
| Inequity in childhood immunization in India: a systematic review. | 2012 | Mathew, Joseph L | RAYYAN-EXCLUSION-REASONS: not a systematic review, as defined by DARE | Wrong study design |
| Reasons related to non-vaccination and under-vaccination of children in low and middle income countries: findings from a systematic review of the published literature, 1999-2009. | 2011 | Rainey, Jeanette J and Watkins, Margaret and Ryman, Tove K and Sandhu, Paramjit and Bo, Anne and Banerjee, Kaushik | RAYYAN-EXCLUSION-REASONS: contains intervention studies | Wrong outcome |
| Factors affecting the uptake of vaccination by the elderly in Western society | 2014 | Eilers R. and Krabbe P.F.M. and de Melker H.E. | RAYYAN-EXCLUSION-REASONS: does not report socioeconomic inequalities in vaccine uptake | Wrong outcome |
| Reasons given for non-vaccination, under-vaccination and delayed vaccination of children and adolescents in sub-Saharan Africa: a systematic review |  | Lauren Perieres, Valerie Seror, Patrick Peretti-Watel, Sylvie Boyer, Cheikh Sokhna | RAYYAN-EXCLUSION-REASONS: does not report the correct socioeconomic inequalities | Wrong outcome |
| Mapping global acceptance of COVID-19 vaccine: a systematic review and meta-analysis |  | Qian Wang, Simeng Hu, Fanxing Du, Shujie Zang, Yuting Xing, Xu Zhang, Zhiqiang Qu, Zhiyuan Hou | RAYYAN-EXCLUSION-REASONS: does not report socioeconomic inequalities in vaccine uptake | Wrong outcome |
| COVID-19 vaccine acceptance and its associated factors in Ethiopia: a systematic review and meta-analysis |  | Birye Dessalegn Mekonnen, Banchigizie Adane Mengistu | RAYYAN-EXCLUSION-REASONS: focus on attitudes/perceptions | Wrong outcome |
| Systematic review and meta-analysis of COVID-19 vaccination acceptance |  | Ruhana Che Yusof, Norhayati Mohd Noor, Mohd Azman Yacob | RAYYAN-EXCLUSION-REASONS: focus on attitudes/perceptions | Wrong outcome |
| Vaccine Hesitancy: Where We Are and Where We Are Going | 2017 | McClure, CC and Cataldi, JR and O'Leary, ST | RAYYAN-EXCLUSION-REASONS: not a systematic review, as defined by DARE | Wrong study design |
| The Uptake of Human Papillomavirus (HPV) Vaccine Among Adolescent Females in the United States: A Review of the Literature | 2011 | Bartlett, JA and Peterson, JA | RAYYAN-EXCLUSION-REASONS: does not report socioeconomic inequalities in vaccine uptake | Wrong outcome |
| A rapid review of evidence on the determinants of and strategies for COVID-19 vaccine acceptance in low- and middle-income countries | 2021 | Moola, S and Gudi, N and Nambiar, D and Dumka, N and Ahmed, T and Sonawane, IR and Kotwal, A | RAYYAN-EXCLUSION-REASONS: focus on attitudes/perceptions | Wrong outcome |
| Parents' Decisions to Vaccinate Children against COVID-19: A Scoping Review | 2021 | Pan, FM and Zhao, HY and Nicholas, S and Maitland, E and Liu, RG and Hou, QZ | RAYYAN-EXCLUSION-REASONS: does not report vaccine uptake | Wrong outcome |
| Human Papillomavirus Vaccine Uptake in Adolescent Boys: An Evidence Review | 2016 | Voss, DS and Wofford, LG | RAYYAN-EXCLUSION-REASONS: not a systematic review, as defined by DARE | Wrong study design |
| Developing evidence for improving childhood vaccine adoption and uptake in low- and middle-income countries: a systematic review | 2022 | Aslam, F and Ali, I and Babar, ZUD and Yang, Y | EXCLUSION-REASONS: does not report socioeconomic inequalities in vaccine uptake | Wrong outcome |
| Barriers to Human Papillomavirus Vaccination Among US Adolescents A Systematic Review of the Literature | 2014 | Holman, DM and Benard, V and Roland, KB and Watson, M and Liddon, N and Stokley, S | RAYYAN-EXCLUSION-REASONS: Mixed review: relevant information cannot be separated from the irrelevant | Wrong outcome |
| Acceptability of and barriers to human papillomavirus vaccination in China: A systematic review of the Chinese and English scientific literature | 2022 | Wang, D and Wu, J and Du, JS and Ong, H and Tang, BW and Dozier, M and Weller, D and Campbell, C | RAYYAN-EXCLUSION-REASONS: focus on attitudes/perceptions | Wrong outcome |
| Access to Vaccination among Disadvantaged, Isolated and Difficult-to-Reach Communities in the WHO European Region: A Systematic Review | 2022 | Ekezie, W and Awwad, S and Krauchenberg, A and Karara, N and Dembinski, L and Grossman, Z and del Torso, S and Dornbusch, HJ and Neves, A and Copley, S and Mazur, A and Hadjipanayis, A and Grechukha, Y and Nohynek, H and Damnjanovic, K and Lazic, M and Papaevangelou, V and Lapii, F and Stein-Zamir, C and Rath, B and ImmuHubs Consortium | RAYYAN-EXCLUSION-REASONS: does not report the correct socioeconomic inequalities | Wrong outcome |
| Canadian school-based HPV vaccine programs and policy considerations | 2017 | Shapiro, GK and Guichon, J and Kelaher, M | RAYYAN-EXCLUSION-REASONS: wrong outcome | Wrong outcome |
| A systematic review of factors affecting vaccine uptake in young children | 2017 | Smith, LE and Amlot, R and Weinman, J and Yiend, J and Rubin, GJ | RAYYAN-EXCLUSION-REASONS: does not report socioeconomic inequalities in vaccine uptake | Wrong outcome |
| Parents' knowledge, beliefs, acceptance and uptake of the HPV vaccine in members of The Association of Southeast Asian Nations (ASEAN): A systematic review of quantitative and qualitative studies | 2021 | Wijayanti, KE and Schutze, H and MacPhail, C and Braunack-Mayer, A | RAYYAN-EXCLUSION-REASONS: Mixed review: relevant information cannot be separated from the irrelevant | Wrong outcome |
| Inequality in the distribution of Covid-19 vaccine: a systematic review. | 2022 | Bayati, Mohsen and Noroozi, Rayehe and Ghanbari-Jahromi, Mohadeseh and Jalali, Faride Sadat | RAYYAN-EXCLUSION-REASONS: does not report vaccine uptake | Wrong outcome |
| Factors Associated With Vaccination Compliance in Southeast Asian Children: A Systematic Review. | 2021 | Kalaij, Ayers Gilberth Ivano and Sugiyanto, Michael and Ilham, Ahmad Fadhil | RAYYAN-EXCLUSION-REASONS: does not report vaccine uptake | Wrong outcome |
| Disparities and reverse disparities in HPV vaccination: A systematic review and meta-analysis. | 2019 | Spencer, Jennifer C. and Calo, William A. and Brewer, Noel T. | RAYYAN-EXCLUSION-REASONS: does not report socioeconomic inequalities in vaccine uptake | Wrong outcome |
| Factors affecting access to immunisation of under-five-year-olds. | 2019 | Wyllie-Schmidt, Cilla and Tipa, ZoÃ« and McClunie-Trust, Patricia | RAYYAN-EXCLUSION-REASONS: Mixed review: relevant information cannot be separated from the irrelevant | Wrong outcome |
| Hpv vaccine adherence among adolescents: integrative review. | 2019 | Calixto de Carvalho, Ayla Maria and Leite Rangel Andrade, Elaine Maria and Tolstenko Nogueira, LÃ­dya and Evangelista de AraÃºjo, Telma Maria | RAYYAN-EXCLUSION-REASONS: does not report vaccine uptake | Wrong outcome |
| Vacunaciã³n contra Hepatitis B: un estudio de revisiã³n. | 2017 | de AraÃºjo, Telma Maria Evangelista and de Sousa, Karinna Alves Amorim and Soares Dias, Samya Raquel and Cavalcante Oliveira, Vanessa and Bastos Marques, Evellyn Stefanne | RAYYAN-EXCLUSION-REASONS: Mixed review: relevant information cannot be separated from the irrelevant | Wrong outcome |
| Human Papillomavirus Vaccine Uptake, Knowledge, and Acceptance for Youth: A Systematic Review of Appalachia. | 2018 | Ryan, Chelsea and Duvall, Kathryn L. and Weyant, Emily C. and Johnson, Kiana R. and Wood, David | RAYYAN-EXCLUSION-REASONS: does not report socioeconomic inequalities in vaccine uptake | Wrong outcome |
| Postawy i edukacja wakcynologiczna rodzicã³w. | 2018 | DoÅ„ka, Katarzyna and SuwaÅ‚a, Marlena and Zarzycka, Danuta and Sobolewska-Samorek, Agnieszka and PaÅºdzior, Violetta | RAYYAN-EXCLUSION-REASONS: Mixed review: relevant information cannot be separated from the irrelevant | Wrong outcome |
| HPV Vaccine Uptake Among Canadian Youth and The Role of the Nurse Practitioner. | 2016 | Scott, Katlyn and Batty, Mary | RAYYAN-EXCLUSION-REASONS: does not report the correct socioeconomic inequalities | Wrong outcome |
| The Impact of COVID-19 Pandemic on Inequity in Routine Childhood Vaccination Coverage: A Systematic Review | 2022 | Spencer, N., Markham, W., Johnson, S., Arpin, E., Nathawad, R., Gunnlaugsson, G., Homaira, N., Rubio, M.L.M., Trujillo, C.J. | RAYYAN-EXCLUSION-REASONS: wrong outcome | Wrong outcome |
| A scoping review of literature exploring factors affecting vaccine uptake within Roma communities across Europe | 2022 | Cronin, A., Ibrahim, N. | RAYYAN-EXCLUSION-REASONS: Mixed review: relevant information cannot be separated from the irrelevant | Wrong outcome |
