# Supplementary material for: Socioeconomic inequalities in vaccine uptake: A global umbrella review
Source: PLoS One. 2023 Dec 13;18(12):e0294688. doi: 10.1371/journal.pone.0294688 (PMC10718431; doi:10.1371/journal.pone.0294688)
Supplement: S7 Appendix — (DOCX) [file pone.0294688.s007.docx]

**S7 Appendix:** Characteristic of included studies.

| **Author (year)**  **Funding** | **Relevant studies** (total)  **Search Date** | **Vaccine/s**  **Uptake** | **Location** | **Population** | **SE Inequalities** |
| --- | --- | --- | --- | --- | --- |
| **Ali** (2022)  *Narrative synthesis Meta-analysis*  **Funding:**  Gavi, the Vaccine  Alliance, and by the Bill Melinda Gates Foundation | **Relevant studies:**  87 narratively synthesised, 22 of which were included in the meta-analysis (108)  **Search date:**  June 15^th^ 2021 (end of publication restriction) | BCG^a^, OPV/IPV^b^, MCV^c^, DTP^d^ **(EPI^e^ 1974)** EPI + Hep B^f^; EPI + Hep B, MMR; EPI + Hep B, JE^g^; EPI + Hep B, Hib^h^; EPI + Hep B, Hib, YF^i^; EPI + Hep B, Hib, MMR; DTP, MCV, BCG; DTP; MCV; IPV/OPV; Hep; Influenza; Other; Not given  **Uptake:**  Unspecified^1^ | **Low and middle income countries:**  Afghanistan, Bangladesh, Brazil, Cambodia, Cameroon, China, Eswatini, Ethiopia, Gambia, Ghana, India, Indonesia, Kenya, Kyrgyz Republic, Laos, Madagascar, Malawi, Mongolia Mozambique, Myanmar, Namibia, Nepal, Nigeria, Pakistan, South Africa, Tanzania, Thailand, Togo, Uganda, Various (unspecified), Vietnam, Zambia | **General**  Eligible children, adolescents and adults | Wealth  Occupation  Maternal education |
| **Arat** (2019)  *Narrative synthesis*  **Funding:**  European Commission | **Relevant studies:**  15 (15 articles, 14 studies)  **Search date:**  July 20^th^ 2017 | DTP MMR  **Uptake:**  Undefined^2^ | **EEA/EFTA countries and Australia:** Australia, Belgium, France, Germany, Greece, Ireland, Italy, Netherlands, Spain, Sweden, UK | **General**  Children under 5 years | Parental income  Education  Occupation  Area-level SES |
| **Bocquier** (2017)  Narrative synthesis  **Funding:**  Agence Nationale de Sécurité du  Médicament et des Produits de Santé (ANSM) and the Agence Nationale  de la Recherche (ANR). | **Relevant studies:**  34 (43 articles, 41 studies)  **Search date:**  April 12^th^ 2016 (end of publication restriction) | **Series:**  DTP, Polio, Hib;  DTP, Polio, Hib, MMR;  DTP, Polio, Hib, Varicella;  DTP, Polio, Hib, PCV^j^, MenC; PCV; Men C; Varicella; Influenza; Rotavirus; Hep B; Hep A; Polio; Pertussis; Hib  **Uptake:**  Individual vaccine uptake  Series completion | **Developed countries:** US, Canada, Belgium, UK, Ireland, Germany, Greece, Italy, Australia | **General**  Children under 12 years | Parental income Education  Occupation  Combination of the above |
| **Desalew** (2020)  *Meta-analysis*  **Funding:**  No financial support declared. | **Relevant studies:**  28 (38)  **Search date:**  2020 (end of publication restriction) | **Series (EPI 1974):**  DTP, Polio, Measles, BCG  **EPI 2004:** Hep B, Hib, PCV, Rotavirus  **Uptake:**  Initiation  Completion | Ethiopia | **General**  Children aged 12-23 months | Maternal education Maternal occupation Wealth status Husband employment |
| **Do** (2021)  *Narrative synthesis*  **Funding:**  National Cancer Institute | **Relevant studies:**  11 (25)  **Search date:**  February 2019, updated February 2020 | HPV  **Uptake:**  Initiation  Completion = 3 doses  Missed opportunities^k^ | USA | **General**  Adolescent (unspecified age) males and females. | Area-level poverty |
| **Dyda** (2016)  *Narrative synthesis*  *Meta-analysis*  **Funding:**  PhD scholarship National Health and  Medical Research Council | **Relevant studies:**  2 (22)  **Search date:**  May 31^st^ 2015 (end of publication restriction) | Influenza Pneumococcal  **Uptake:**  Unspecified | Australia | **General**  Adults ≥65 and <65 | Education  Income  Meta-analysis conducted, but SE inequalities reported in narrative format. |
| **Eshete** (2020)  *Meta-analysis*  **Funding:**  No financial support declared. | **Relevant studies:**  30 (30)  **Search date:**  April 2019 – August 2019, updated January 20^th^ 2020 | **Series (EPI 974):** DTP, Polio, Measles, BCG  **Epi 2004:** Hep B, Hib, PCV, Rotavirus  **Uptake:**  Incompletion  Completion  x1 BCG, x3 Penta (DTP, Hep B, Hib), x3 Polio, x3 PCV, x2 Rotavirus, x1 Measles | Ethiopia | **General**  Children 12-23 months | Maternal education |
| **Fernandez** (2015)  *Narrative synthesis*  **Funding:**  Not provided. | **Relevant studies:**  16 (23)  **Search date:**  April 2014 (end of publication restriction) | HPV^l^  **Uptake:**  Initiation (1/2 doses)  Completion (3 doses) | **Europe:** Netherlands, Sweden, Denmark, Belgium, Italy, France, Germany, UK, Scotland, Greece | **General**  Eligible females, no age restriction | Education  Deprivation  Employment  Parental income |
| **Fisher** (2013)  *Meta-analysis*  **Funding:**  Centre for the  Development and Evaluation of Complex  Interventions for Public Health Improvement  (DECIPHer) | **Relevant studies:** 19 (29 articles, 27 studies)  **Search date:**  March 9^th^ 2012 (end of publication restriction) | HPV  **Uptake:**  Initiation  Completion | **Not specified:** USA, Belgium, Netherlands, Canada, UK | **Females**  ≤ 18 years | Income/area level deprivation Education |
| **Forshaw** (2017)  *Meta-analysis*  **Funding:**  PhD scholarship National Institute for Health Research | **Relevant studies:**  37 (37)  **Search date:**  June 29^th^ 2016 | **Series (EPI 974):** DTP, Polio, Measles, BCG  **EPI (2004):**  Hep B, Hib, PCV, Rotavirus  **Uptake:**  Initiation  Completion | **Global:** Iraq, Ethiopia, Nigeria, Uganda, Brazil, USA, Kenya, India, Greece, Bangladesh, Malawi, Mali, Belgium, Zimbabwe, Burkina Faso, Zambia, Indonesia, Vietnam, Turkey, Cameroon | **General**  Mothers with children under 12 years | Maternal education |
| **Galadima** (2021)  *Narrative synthesis*  **Funding:**  No financial support declared. | **Relevant studies:**  15 (51)  **Search date:**  October 26^th^ 2020 (end of publication restriction) | **Series (EPI):**  BCG, OPV, Hep B, DTP, Measles, YF  **Uptake:**  Unspecified, any | **Africa:** Angola, Burkina Faso, Cameroon, Congo, Ethiopia, Gambia, Ghana, Kenya, Mozambique, Nigeria Tanzania, Uganda | **General**  Children under 5 years | Parental education Maternal occupation Income |
| **Galbraith** (2016)  *Narrative synthesis*  **Funding:**  No financial support declared. | **Relevant studies:**  4 (67)  **Search date:**  January 2015 (end of publication restriction) | HPV  **Uptake:**  Initiation  Completion | USA | **African Americans and/or Latinos** Female caregivers of females aged 10-19 years | Poverty Income Education |
| **Gallagher** (2016)  *Narrative synthesis*  **Funding:**  Medical Research Council, UK, Instituto de Salud Carlos III, Agència de Gestió d’Ajuts Universitaris i de Recerca, and European Community’s Seventh Framework Programme | **Relevant studies:**  14 (61)  **Search date:**  February 2014 | **Adolescent schedule if not given prior to aged 10:**  DTP; HPV; Men conjugate; Influenza; Hep A; Hep B; MMR; Tick borne encephalitis; JE; Typhoid; Cholera; Rabies; Varicella  **Uptake:**  Initiation  Completion  *Only DTP, HPV, and influenza were analysed.* | **No restrictions:** Canada, USA, France, Various (unspecified), Australia, UK, Greece, Peru | **General**  Adolescents, aged 9-19 years | Median neighbourhood/parental income  Average adult education Poverty status  Maternal education |
| **Kessels** (2012)  *Narrative synthesis*  **Funding:**  Australian Research Council Linkage  Grant Project | **Relevant studies:**  11 (33 articles, 25 studies)  **Search date:**  March 7^th^ 2011 (end of publication restriction) | HPV  **Uptake:**  Initiation  Completion | **No restrictions:** USA, Canada, Australia, UK, Netherlands, France | **General**  Adolescents, eligible females aged 9-18 years | Parental education Family income |
| **Loke** (2017)  *Narrative synthesis*  **Funding:**  No financial support declared. | **Relevant studies:**  7 (42)  **Search date:**  March 4^th^ 2017 (end of publication restriction) | HPV  **Uptake:**  First dose (initiation)  Third dose (completion) | **Unspecified:**  USA, UK, Norway, The Netherlands, Germany, France, Denmark, Latvia, Hong Kong, Taiwan, Malaysia, Japan, Canada, Australia | **General**  Adolescents, males, and females | Maternal education |
| **Lucyk** (2019)  *Narrative synthesis*  **Funding:**  University of  Calgary and the Alberta Ministry of Health | **Relevant studies:**  22 (42)  **Search date:**  May 2017 (end of publication restriction) | Influenza  (Seasonal and pandemic)  **Uptake:**  Undefined | **High-income countries:** USA, Canada, Denmark, Belgium, South Korea, Japan, Germany, Australia, Israel, New Zealand, UK, Italy, Ireland, Poland, Spain | **General**  No restrictions | Education  Occupational class  Income/poverty |
| **Mansfield** (2021)  *Narrative synthesis*  **Funding:**  National Institute of Nursing Research of the National  Institutes of Health | **Relevant studies:**  5 (57)  **Search date:**  January 2020 | HPV  **Uptake:**  Initiation  Completion | USA | **General**  Parents of, or adolescents, aged 9-18, males and females | Socioeconomic status  Poverty status |
| **Murfin** (2018)  *Narrative synthesis*  **Funding:**  Not provided. | **Relevant studies:**  6 (10)  **Search date:**  June 13^th^ 2018 | HPV  **Uptake:**  Initiation  Completion | **Developed countries:** Norway, USA, Germany | **General**  Eligible females | Education Income Occupation |
| **Nagata** (2011)  *Narrative synthesis*  **Funding:**  Initiative for  Vaccine Research and the Social Determinants of Health Unit at the World  Health Organization | **Relevant studies:**  10 (58)  **Search date:**  January 2011 | Influenza  **Uptake:**  Undefined | **Unspecified:**  Asia, Europe, Latin America, Middle-East, various (unspecified) | **General**  Adults < 65 | Education Socioeconomic status Deprivation |
| **Okoli** (2020)  *Narrative synthesis*  **Funding:**  GlaxoSmithKline, Merck, Sanofi  Pasteur, Pfizer and Roche-Assurex | **Relevant studies:**  20 (34)  **Search date:**  January 2018, updated January 7^th^ 2020 | Influenza  **Uptake:**  Undefined | **Not specified:**  Spain, USA, UK, Europe, China, Israel, Italy, Ireland, France, Australia, Thailand, Canada, South Korea, Switzerland, Singapore, Serbia, Japan | **General**  Adults ≥65 years | Household income  Education  Social class  Employment |
| **Schellenberg** (2020)  *Narrative synthesis*  **Funding:**  Not provided. | **Relevant studies:**  8 (12)  **Search date:**  October 2019 (end of publication restriction) | **Vaccination status:**  MMR; Varicella; DTP; Hib; Meningococcal; PCV; Rotavirus; Hep B  **Uptake:**  Initiation  Completion | Canada | **General**  Children, aged ≤ 7 years | Household income Parental education  Unemployment rate |
| **Shenton** (2020)  *Narrative synthesis*  *Scoping review*  **Funding:**  National Institute of Allergy And  Infectious Diseases of the National Institutes of Health | **Relevant studies:**  83% (125)  Percentages of relevant studies were provided, instead of exact numbers.  **Search date:**  December 31^st^ 2018 (end of publication restriction) | **Routine vaccination, EPI schedule 1974:**  BCG; DTP; Polio; Measles  **2004:**  Hep B; Hib; Rubella; PCV; Rotavirus  **Uptake:**  Completion | **Demographic and Health Survey countries:**  Malawi, India, Kazakhstan, Nepal, Vietnam, Nigeria, Bangladesh, Philippines,  Indonesia, Burkina Faso, Cambodia, Tanzania, Kenya, Nepal, Uganda, Burundi, Pakistan, Madagascar, Ethiopia, Bolivia, Ghana, Zimbabwe, Benin, Senegal, DRC, Afghanistan, Various (unspecified) | **General**  Children, aged > 60 months | Maternal education Wealth index Paternal education |
| **Shin** (2022)  *Narrative synthesis*  **Funding:**  Ministry of Education | **Relevant studies:**  14 (30)  **Search dates:**  July 2020 (end of publication restriction) | HPV  **Uptake:**  Initiation | **No restrictions:**  USA, Denmark | **General**  Eligible boys/men | Parental educational level  Parental employment status  Household income |
| **Tauil** (2016)  *Narrative synthesis*  **Funding:**  PhD scholarship Coordination for the Improvement of Higher Education Personnel (CAPES)/São Paulo Research Foundation | **Relevant studies:**  10 (23)  **Search dates:**  July 17^th^ 2014 – July 21^st^ 2014 | **Routine vaccination:**  DTP (x3); Polio (x3); Measles (x1)  **Uptake:**  Incompletion  Completion | **Global:** Burkina Faso, Mozambique, Kenya, Philippines, Brazil, Belgium, Canada, USA | **General**  Caregivers of children, aged ≤ 24 months | Maternal education Socioeconomic status Mother working inside/outside the home |
| **Tilahun** (2020)  *Scoping Review*  **Funding:**  Alliance for Health  Policy and Systems Research | **Relevant studies:**  15 (55)  **Search dates:**  November 28^th^ 2018 | **Routine vaccination (EPI programme of Ethiopia):**  DTP, Polio, Measles, BCG  **Uptake:**  Completion | Ethiopia  (National and regional) | **General** Children, aged ≤ one year | Household economic status Caregiver/mother’s education Caregiver/mother’s occupation |
| **Wang** (2018)  Meta-analysis  **Funding:**  Chinese National Natural Fund, Science Technology  Demonstration Project for Emerging Infectious Diseases Control and Prevention, Jiangsu Provincial Six Talent Peak, Jiangsu Provincial Key Medical Discipline | **Relevant studies:**  25 (126)  Cannot specifically identify which relevant studies are included.  **Search dates:**  March 18^th^ 2018 | Influenza  **Uptake:**  Undefined | Mainland China | **General**  Not specified | Education |

| 1Unspecified – no restrictions were placed on the measure of uptake.  2Undefined – there was no mention of eligible measures of uptake.  a BCG - Bacillus Calmette-Guérin vaccine, protecting against Tuberculosis.  b OPV/IPV – Oral Poliovirus vaccine/inactivated poliovirus vaccine.  c MCV – Measles-containing vaccine.  d DTP – Diphtheria-Tetanus-Pertussis vaccine.  e EPI – Expanded Programme on Immunisation.  f Hep B – Hepatitis B vaccine.  g JE – Japanese Encephalitis vaccine.  h Hib – Haemophilus Influenzae type B vaccine.  i YF – Yellow Fever vaccine.  j PCV – Pneumococcal vaccine.  k Missed opportunities – A clinical encounter when at least one adolescent vaccination was received, where another vaccine could have been administered as well.  l HPV – Human Papillomavirus vaccine. |
| --- |
